# Association of nociplastic pain with executive function decline in a longitudinal cohort of middle-age adults: a prospective cohort study

**DOI:** 10.1016/j.bja.2025.08.002

**Published:** 2025-08-20

**Authors:** Eoin M. Kelleher, Xin-You Tai, Andrew Schrepf, Trishna Rathod-Mistry, Thomas Nichols, Irene Tracey, Anushka Irani

**Affiliations:** 1 Nuffield Department of Clinical Neurosciences, University of Oxford, Oxford, UK; 2 Department of Anesthesia, Critical Care and Pain Medicine, Massachusetts General Hospital, Boston, MA, USA; 3 Chronic Pain and Fatigue Research Centre, University of Michigan, Ann Arbor, MI, USA; 4 Nuffield Department of Orthopaedics, Rheumatology and Musculoskeletal Sciences, University of Oxford, Oxford, UK; 5 Big Data Institute, Nuffield Department of Population Health, University of Oxford, Oxford, UK; 6 Division of Rheumatology, Mayo Clinic Florida, Jacksonville, FL, USA

**Keywords:** chronic pain, cognition, epidemiology, executive function, nociplastic pain, pain, UK Biobank

## Abstract

**Background:**

Patients with chronic pain, frequently report cognitive problems (‘brain fog’), particulary those with nociplastic pain, which involves dysregulated pain processing. However, the association with objective executive dysfunction and decline over time is unclear. We examined whether nociplastic pain severity is associated with executive dysfunction and decline, and explored which factors, such as sex or associated non-pain symptoms, mediate this relationship.

**Methods:**

We conducted a prospective cohort study using UK Biobank. Baseline cognitive assessments (2017–20), a pain questionnaire (2019), and follow-up cognitive assessments (2021–2) were analysed. Nociplastic pain severity was measured using the fibromyalgia index (0–31), a continuous measure of widespread pain and symptom severity. A latent executive function measure was derived using confirmatory factor analysis. Cross-sectional associations were examined using linear regression, and longitudinal structural equation modelling assessed cognitive decline. Mediation analysis evaluated indirect effects of abnormal sleep duration, anxiety, depression, brain fog, fatigue, and pain qualities.

**Results:**

We included 35 423 participants (median age 65 yr; 52% female). Greater nociplastic pain severity was associated with worse executive function cross-sectionally (β=−0.49 centiles per 1 point increase in fibromyalgia index; 95% confidence interval −0.59 to −0.39), with a stronger effect in males. However, nociplastic pain severity was not associated with a faster rate of executive function decline. Abnormal sleep duration, anxiety, pain intensity, and neuropathic pain features partially mediated the cross-sectional association.

**Conclusions:**

Nociplastic pain severity is associated with worse executive function but reassuringly does not suggest worse cognitive decline. Abnormal sleep duration, anxiety, and pain intensity are highlighted as potential intervention targets.

**Editor’s key points**

• Few studies have assessed the association between nociplastic pain such as fibromyalgia and impairment in executive function (e.g. flexibility, inhibitory control, working memory).
• Based on the large UK Biobank cohort, authors find a cross-sectional association between nociplastic pain severity and worse executive function, particularly in males, which is partially accounted for by sleep and anxiety disorders. In contrast nociplastic pain does predict a decline in executive function over time.
• This finding does not exclude reverse causation, i.e. that a decline in cognitive function predicts nociplastic pain.

Cognitive impairment is a commonly reported but poorly understood symptom of chronic pain conditions, such as fibromyalgia. Patients frequently report difficulties in cognitive functions, sometimes referred to as ‘brain fog’, yet the relationship with objective executive function remains unclear. Executive function refers to a set of higher-order cognitive processes that support goal-directed behaviour. These include working memory (the ability to hold and manipulate information over short periods), cognitive flexibility (the capacity to shift perspectives or strategies in response to changing demands), and inhibitory control (the ability to suppress impulses or irrelevant responses).^1^ Although studies have reported an association between chronic pain and memory decline and dementia,^2^ evidence for an association with objective executive dysfunction is mixed, as is the relationship with decline in executive function over time. While some cross-sectional studies and meta-analyses report impairments in working memory, task switching, and inhibitory control,^3^, ^4^, ^5^ others find minimal or no deficits once mood, fatigue, or sleep disturbance are accounted for.^6^^,^^7^ Longitudinal data are also inconsistent, with some studies showing accelerated cognitive decline in pain populations, while others report domain-specific or null effects.^2^^,^^8^

The International Association for the Study of Pain (IASP) categorises chronic pain b underlying mechanisms into nociceptive, neuropathic, and nociplastic pain.^9^ Nociplastic pain, the most recently defined, arises from altered nociception without evidence of peripheral nociceptor activation or somatosensory system damage, and is thought to underlie conditions such as fibromyalgia, chronic tension-type headache, and irritable bowel syndrome, where cognitive difficulties are frequently reported by patients.^9^

These nociplastic pain disorders are characterised by a symptom cluster consisting of sleep difficulties, pain, affect (depression/anxiety), cognitive complaints (brain fog), and low energy (fatigue). These have been termed ‘SPACE’ symptoms.^10^ We do not know what factors contribute to objective cognitive dysfunction in chronic pain, but these may be likely candidates. Experimental studies suggest that disrupted or insufficient non-rapid eye movement (non-REM) sleep impairs sustained attention and cognitive flexibility, particularly through increased reaction time variability and attentional lapses,^11^^,^^12^ and may contribute to fibromyalgia-like symptoms and broader cognitive complaints.^13^ In UK Biobank, both short and long sleep durations demonstrate U-shaped relationships with cognitive performance and decline.^14^ Furthermore, pain characteristics, such as intensity and bodily distribution (e.g. multisite or widespread pain), may also influence cognitive dysfunction by consuming attentional resources.^15^ Analgesics, such as opioids,^16^^,^^17^ gabapentinoids,^18^ and tricyclic antidepressants (TCAs),^19^ may also play a role in cognitive dysfunction in chronic pain.

Sex differences in pain and cognition have also been observed in both preclinical models^20^ and human neuroimaging studies,^21^ making sex a potential moderator of this relationship.

To address these gaps, we conducted the first large-scale, longitudinal study of nociplastic pain severity and executive function in UK Biobank, a deeply phenotyped cohort of middle-aged adults. We examined whether nociplastic pain is associated with executive dysfunction, predicts cognitive decline over time, and whether these associations differ by sex. We focused on executive functions because UK Biobank tasks better capture these domains than memory, and executive dysfunction in chronic pain remains underexplored despite its relevance to daily functioning. We also assessed whether SPACE symptoms, pain characteristics, or analgesic use mediate these relationships, to identify modifiable intervention targets.

## Methods

### Study design

This was a longitudinal cohort study set within UK Biobank, a population-based cohort of ∼500 000 British adults aged 40–69 yr, recruited between 2006 and 2010.^22^ Participants attended baseline assessment centres where sociodemographic, lifestyle, and health data were collected. Follow-up included online questionnaires and assessment visits, with an expanded cognitive assessment introduced in 2016, described below. The main UK Biobank study received ethical approval from the NHS National Research Ethics Service (Ref. 11/NW/0382), and this study was approved under application 45465.

### Study population

Participants were eligible if they completed a cognitive assessment in 2017–20, following the December 2016 introduction of the expanded UK Biobank battery, and an online pain questionnaire in 2019, which included the fibromyalgia index (FMI). To evaluate longitudinal changes, those with a follow-up cognitive assessment in 2021–2 were included. Individuals with dementia or serious neurological conditions at the time of cognitive assessment were excluded (Supplementary Table S1). Because of the UK Biobank protocol, cognitive and pain assessments were conducted at separate time points, leading to variable time intervals between them (Supplementary Fig. S1). Sensitivity analyses stratified by timing were performed (see below). UK Biobank field IDs and a timeline of data collection are provided in Supplementary Table S1 and Supplementary Figure S2, respectively.

### Pain assessment

Chronic pain was defined as pain persisting for >3 months,^9^ assessed via the 2019 pain questionnaire. The FMI, derived from the 2016 Fibromyalgia Survey Criteria, which combines the widespread pain index (WPI, 0–19) and symptom severity scale (SSS, 0–12), creating a continuous measure (0–31) where higher scores indicate more severe nociplastic pain.^23^ The FMI was analysed as a continuous measure of nociplastic pain features and, to ensure appropriate interpretation, associations were stratified by chronic pain status. The FMI has demonstrated predictive value for pain outcomes in the general population after surgery.^24^ Neuropathic pain features were assessed using the Douleur Neuropathique 4 (DN4) questionnaire, evaluating burning, tingling, and electric shock-like pain sensations. The DN4 was offered only in participants reporting chronic pain, and was included to examine whether neuropathic pain qualities mediated the association between nociplastic pain and executive function.

### Cognitive assessment

Cognitive function was assessed using three unsupervised, touchscreen-based tasks administered as part of the UK Biobank imaging study’s expanded cognitive battery. This enhanced assessment included tests targeting multiple cognitive domains, including executive function, processing speed, attention, and abstract reasoning, among others.^25^ The specific tasks used in the present study were:

• Trail making test (TMT): alternating between numbers and letters in ascending order (e.g. 1-A-2-B) to measure executive function.
• Digit-symbol substitution (SDS): matching symbols to digits using a reference grid within 60 s, assessing processing speed and attention.
• Matrix pattern recognition: selecting the missing piece to complete a visual pattern, measuring abstract reasoning and executive components such as rule tracking, cognitive flexibility, and visual–spatial working memory.

These tests form part of a broader UK Biobank battery that also includes assessments of memory (e.g. pairs matching, numeric memory), verbal reasoning (e.g. fluid intelligence), and vocabulary knowledge (e.g. picture vocabulary).^26^ The trail making score was calculated as the time taken to complete the alphanumeric task minus the numeric-only version, indexing executive control.^14^ All test scores were age-adjusted using polynomial regression models and rescaled using proportion of maximum scaling. TMT scores were reverse coded so higher values indicated better performance. See Supplementary material (Appendix A) for full preprocessing details.

### Confounders

Confounders were selected *a priori* based on known associations between nociplastic pain and cognition,^27^ and included age, sex, ethnicity, socioeconomic deprivation (Townsend Index, a measure of material deprivation based on unemployment, car ownership, home ownership, and household overcrowding^28^), education level, smoking status, and body mass index (BMI).

### Mediators

Three groups of mediators were assessed. (1) SPACE Cluster^10^: sleep duration (<7, 7–9, >9 h),^29^ pain intensity (0–10 numeric rating scale), depressive symptoms (Patient Health Questionnaire 9-item, PHQ-9), anxiety symptoms (Generalized Anxiety Disorder 7-item, GAD-7), cognitive symptoms (subjective cognitive difficulties from SSS), and fatigue (fatigue severity scale, FSS). Of note, this was only offered to participants who reported fatigue on the SSS; participants who did not report fatigue were given a score of 0. (2) Pain characteristics: pain intensity, widespread pain (WPI), and neuropathic pain features (DN4). (3) Analgesia use: self-reported use of opioids, TCAs, and gabapentinoids from 2017 to 2020 interviews. Self-reported prescription medications were extracted from verbal interviews conducted by trained nurses during UK Biobank baseline visits. Participants provided medication names, which were subsequently coded by UK Biobank.^30^ We identified and grouped analgesics *a priori* into three classes based on their known cognitive effects and use in pain management: opioids, gabapentinoids, and TCAs. A full list of included codes is provided in Supplementary Table S2.

### Statistical analysis

Baseline characteristics were summarised using means (standard deviation, sd) for continuous variables and frequencies (%) for categorical variables.

### Confirmatory factor analysis

Confirmatory factor analysis (CFA) was conducted to derive a latent measure for executive function from the three cognition tasks (TMT, SDS, matrix pattern recognition). This approach allows the representation of multiple related measures simultaneously while reducing measurement error,^31^^,^^32^ and aligns with previous applications in UK Biobank which demonstrated good correlation with gold standard neuropsychological assessments.^14^^,^^33^ Good model fit was defined as comparative fit index (CFI) and Tucker-Lewis index (TLI) >0.90, and root mean square error of approximation (RMSEA) <0.05. Further details of CFA are in the Supplementary material (Appendix A). To aid interpretation, cognitive outcomes were transformed into centile ranks, where the first centile is the worst performer, and the 100th centile is the best performer.^34^

Longitudinal CFA estimated two latent constructs of executive function at baseline and at follow-up. Factorial invariance across time points was assessed comparing four CFA models (configural, weak, partial strong, and strong invariance) with progressively stricter constraints on the estimated standardised factor loadings and residual variances. Incremental changes in fit indices were used to determine invariance between models.^35^ A threshold of ≤0.01 for ΔCFI and ΔTLI,^36^ ≤0.015 for ΔRMSEA,^37^ and ≤0.03 for ΔSRMR^38^ was used to assess invariance between models. Further details are in the Supplementary material (Appendix B). This ensured that observed changes in executive function over time are as a result of true differences in the underlying construct rather than shifts in measurement properties.^31^ This is particularly important in the context of cognition, where changes may be attributable to evolving characteristics of the tests over time, as opposed to true underlying cognitive shifts.^39^

### Cross-sectional and longitudinal analysis

Linear regression examined the cross-sectional association between FMI and executive function, adjusted for confounders. Analyses were stratified by chronic pain status to account for overlap between FMI symptoms and those in individuals without chronic pain. Two models were assessed. (1) Minimally adjusted: age, sex, and days between pain and cognitive assessments. (2) Fully adjusted: further adjusted for socioeconomic deprivation, ethnicity, education, smoking status, and BMI.

Secondary analyses explored sex differences within the chronic pain group. Interaction terms between sex and chronic pain status were assessed via the likelihood ratio test.

Within the chronic pain group, the relationship between the FMI at baseline and change in executive function at follow-up, controlling for baseline executive function, was investigated using longitudinal structural equation modelling (SEM).^31^

### Mediation analysis

SEM estimated the direct and indirect effects of mediators on the FMI–executive function relationship at baseline and follow-up within the chronic pain group.^40^^,^^41^ Significance was tested using bias-corrected bootstrapping with 5000 resamples, which accommodates non-normal distributions of indirect effects.^42^

Three mediation models examined: (1) SPACE symptoms (sleep, pain, affect, cognition, energy/fatigue); (2) pain characteristics (pain severity, widespread pain, neuropathic pain features); and (3) analgesia use (opioids, TCAs, gabapentinoids).

Covariances between mediators and standardised coefficients for the direct and indirect effects were estimated.

### Sensitivity analyses

First, each cognitive task (TMT, SDS, matrix) was analysed separately to assess its individual contribution to the overall association. Second, participants were stratified based on whether pain data were collected before or after cognitive testing to assess potential temporal influences. Third, analyses were performed by categorising participants into 1-yr intervals between pain and cognition assessments to explore potential trends over time. Finally, the impact of the COVID-19 pandemic on cognitive function was evaluated by comparing participants who completed baseline cognitive assessments before March 2020 with those assessed afterward.

Complete case analyses were performed using R (v4.4.1), using the ‘lavaan’ package for CFA and SEM. Model assumptions were checked. Two-sided *P*-values <0.05 were considered significant. UK Biobank field ID codes are in Supplementary Table S1, and analgesic medication codes in Supplementary Table S2. The study follows STROBE guidelines.

## Results

### Study participants

**Fig 1:**
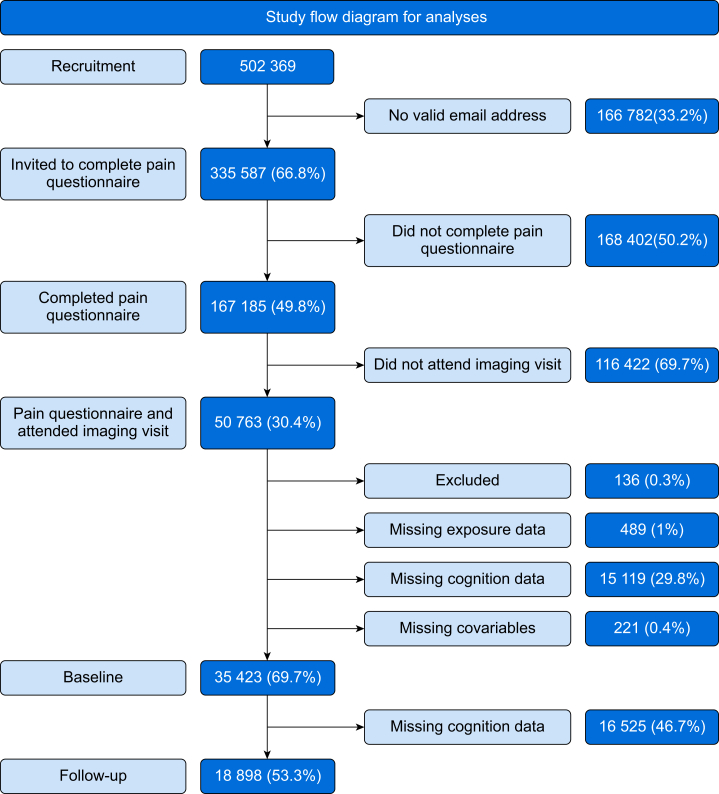
Study flow diagram for UK Biobank participants in cross-sectional and longitudinal analyses of relationship between FMI and executive function. The imaging visit refers to the follow-up imaging visits which began in 2014. All participants for whom UK Biobank had a current e-mail address (∼333 000) were invited to attend follow-up visits and complete online questionnaires. Commencing 2020, UK Biobank began sending postal invitations for the follow-up imaging visit. A small number (<0.5%) of participants have withdrawn or moved outside the UK. An expanded battery of cognitive assessments was introduced in imaging visits conducted after December 2016, and participants who attended the imaging visit before this were not included. The cognitive assessment undertaken during the first imaging visit between 2017 and 2020 was the baseline timepoint for this study. The online pain questionnaire taken in 2019 was taken as the baseline assessment for pain. The online cognitive assessment performed in 2021–2 was the follow-up assessment for this study. For the cross-sectional analysis, participants who completed cognitive tests at the first imaging visit and the experience of pain questionnaire were included. For the longitudinal analysis, participants who subsequently also completed the follow-up online cognitive assessment were included. FMI, fibromyalgia index.

Of 502 369 UK Biobank participants, 335 587 (66.8%) were invited to complete the 2019 pain questionnaire, with 167 185 (49.8%) responding. A total of 35 423 participants who attended the 2017–9 visit were included in the cross-sectional analysis after excluding individuals with missing cognitive data (29.8%), major neurological conditions (0.3%), or incomplete pain/covariate data (1.4%). Of these, 18 898 (53.3%) completed a follow-up online cognitive assessment in 2021–2 (median follow-up: 2.69 yr, range 1.07–4.43 yr) and were included in longitudinal analyses. The population flow diagram is shown in Figure 1.

### Baseline characteristics

Baseline characteristics are summarised in Table 1. The median age of participants was 65 yr (range 44–85 yr), and 52% (*n*=18 588) were female. Chronic pain was reported by 53% of participants (*n*=18 769), with a median of two pain sites (interquartile range 1–2). Compared with participants without chronic pain, those with chronic pain had a significantly higher mean FMI score (5.04, sd 3.91 *vs* 1.96, sd 1.93), higher prevalence of females (56% *vs* 49%), lower prevalence of university educational attainment (50.6% *vs* 55.5%), and had higher mean BMI (26.8 *vs* 25.9 kg m^−2^).

**Table 1:** Baseline characteristics of participants included in cross-sectional association between fibromyalgia index and executive function. Higher values of Townsend Deprivation Index indicate greater social deprivation. Nociplastic pain was assessed using the fibromyalgia index (FMI), with higher scores indicating more severe nociplastic pain. The FMI is the sum of the widespread pain index (WPI) and symptom severity scale (SSS). Pain intensity was measured using numeric rating scale (NRS) among participants who indicated they had chronic pain; higher values indicate more severe pain. Depression was measured using Patient Health Questionnaire 9-item, with higher scores indicating more severe depression symptoms. Anxiety was measured using the General Anxiety Disorder 7-item on the Mental Health and Well-being questionnaire, with higher scores indicating more severe anxiety symptoms. Brain fog was measured using items on subjective cognitive difficulties on SSS, with higher scores indicating more severe brain fog symptoms. Fatigue was measured using the fatigue severity scale (FSS), with higher scores indicating more severe fatigue symptoms. Note the FSS was only offered to participants who reported fatigue on the SSS. Neuropathic pain symptoms measured using the Douleur Neuropathique 4 (DN4), with higher scores indicating more severe neuropathic symptoms.sd, standard deviation; SDS, digit-symbol substitution; TMT, Trail making test.

	Total (*N*=35 423)	No Chronic pain(*N*=16 654)	Chronic pain(*N*=18 769)
Sex, *n* (%)			
Female	18 588 (52)	8085 (49)	10 503 (56)
Male	16 835 (48)	8569 (51)	8266 (44)
Age (yr)			
Mean (sd)	64.5 (7.38)	64.4 (7.43)	64.6 (7.34)
Median (range)	65 (44–85)	65 (44–85)	65 (44–84)
Townsend Deprivation Index			
Mean (sd)	−1.90 (2.73)	−1.94 (2.71)	−1.86 (2.75)
Employment status, *n* (%)			
Employed	12 567 (35)	6155 (37)	6412 (34)
Retired	21 544 (61)	9975 (60)	11 569 (62)
Unemployed/other	1243 (4)	494 (3)	749 (4)
Missing	69 (0.2)	30 (0.2)	39 (0.2)
White ethnicity (%)	97.5	97.6	97.5
University degree (%)	52.9	55.5	50.6
Body mass index (kg m^−2^)			
Mean (sd)	26.4 (4.46)	25.9 (4.13)	26.8 (4.70)
Fibromyalgia index (0–31)			
Mean (sd)	3.59 (3.49)	1.96 (1.93)	5.04 (3.91)
Widespread pain index (0–19)			
Mean (sd)	1.27 (1.97)	0.284 (0.764)	2.14 (2.27)
Symptom severity scale (0–12)			
Mean (sd)	2.32 (2.09)	1.68 (1.64)	2.89 (2.28)
Sleep duration (h)			
Mean (sd)	7.16 (1.02)	7.23 (0.968)	7.10 (1.06)
Sleep duration, *n* (%)			
<7 h	8261 (23)	3373 (20)	4888 (26)
7–9 h	24536 (69)	12061 (72)	12475 (66)
>9 h	2480 (7)	1163 (7)	1317 (7)
Pain intensity, NRS (0–10)			
Mean (sd)	3.70 (2.58)	NA (NA)	3.70 (2.58)
Depression (PHQ-9, 0–27)			
Mean (sd)	5.19 (6.98)	3.28 (5.04)	6.89 (7.96)
Anxiety (GAD-7, 0–21)			
Mean (sd)	1.80 (3.05)	1.40 (2.65)	2.15 (3.32)
Brain fog (SSS)			
Mean (sd)	1.39 (0.575)	1.30 (0.495)	1.47 (0.628)
Fatigue severity scale (FSS)			
Mean (sd)	18.6 (13.2)	15.4 (10.7)	21.4 (14.5)
Douleur Neuropathique 4 (0–7)			
Mean (sd)	1.16 (1.39)	N/A	1.16 (1.39)
Opioid use, *n* (%)	1.13	0.17	1.99
Tricyclic antidepressant use, *n* (%)	1.53	0.54	2.42
Gabapentinoid use, *n* (%)	0.91	0.19	1.56
TMT (s)			
Mean (sd)	34.3 (23.5)	34.0 (23.4)	34.6 (23.6)
TMT, centile rank			
Mean (sd)	50.1 (28.8)	50.6 (28.9)	49.6 (28.8)
SDS, proportion correct			
Mean (sd)	0.954 (0.0934)	0.956 (0.0907)	0.952 (0.0957)
SDS, centile rank			
Mean (sd)	50.0 (28.8)	50.6 (28.6)	49.5 (29.1)
Matrix pattern test, proportion correct			
Mean (sd)	0.597 (0.166)	0.604 (0.165)	0.590 (0.167)
Matrix pattern test, centile rank			
Mean (sd)	50.0 (28.9)	51.3 (28.8)	48.9 (28.9)

Mental health characteristics were worse in the chronic pain group, with higher depression (PHQ-9: 6.89 *vs* 3.28), anxiety (GAD-7: 2.15 *vs* 1.40), and fatigue (FSS: 21.4 *vs* 15.4) scores. Participants with chronic pain reported greater analgesic use, including opioids (2.0% *vs* 0.2%), tricyclic antidepressants (2.4% *vs* 0.5%), and gabapentinoids (1.6% *vs* 0.2%).

Participants with chronic pain in the longitudinal analysis exhibited similar characteristics (Supplementary Tables S3 and S4). Raw cognitive test scores are given in Supplementary Table S5.

### Confirmatory factor analysis

The CFA model estimating a latent factor of executive function displayed excellent model fit (CFI 1.0, TLI 1.0, and RMSEA <0.001; Supplementary Fig. S2).

### Cross-sectional analyses

**Fig 2:**
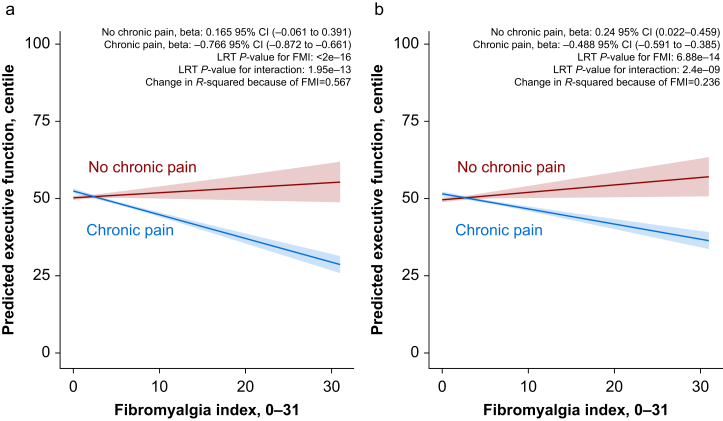
Chronic pain moderates the relationship between fibromyalgia index and executive function. (a and b) Stronger negative association between nociplastic pain severity and executive function in individuals with chronic pain. Panels depict predicted executive function (EF) centile scores across fibromyalgia index (FMI) levels, stratified by the presence or absence of chronic pain. Panel a adjusts for age, sex, and assessment order, while panel b includes additional adjustments for ethnicity, socioeconomic status, education, body mass index, and tobacco use. Centile scores represent an individual’s performance relative to the general population, with a score of 50 corresponding to the median, higher scores indicating better EF, and lower scores indicating poorer EF. Lines represent estimated associations, with shaded regions indicating 95% confidence intervals. A significant interaction between FMI and chronic pain is observed, with stronger negative associations in individuals with chronic pain, as indicated by the interaction *P*-values from likelihood ratio tests (LRT). These findings suggest that increasing FMI scores are associated with greater EF reductions in those experiencing chronic pain.

Greater nociplastic pain severity was associated with worse executive function cross-sectionally (β=−0.49 centiles per unit increase in FMI; 95% confidence interval [CI] −0.59 to −0.39). In the minimally adjusted model, higher FMI scores were associated with worse executive function in individuals with chronic pain (−0.766 centiles per unit increase in FMI; 95% CI −0.872 to −0.661; Fig. 2a) but not in those without chronic pain (0.165 centiles per unit increase in FMI; 95% CI −0.061 to 0.391). These associations attenuated after full adjustment for confounders but remained statistically significant (chronic pain: −0.488 centiles per unit increase in FMI; 95% CI −0.591 to −0.385; Fig. 2b). Detailed results of linear regression are given in the Supplementary material (Appendix C and Supplementary Tables S6–10).

The association between FMI and executive function was stronger in males than females (likelihood ratio tests [LRT] *P*-interaction <0.001). In fully adjusted models, each 1 point FMI increase was associated with a mean 0.76 centile decrease in executive function in males (95% CI −0.929 to −0.588, *P*<0.001; Supplementary Fig. S3), compared with a 0.36 centile decrease in females (95% CI −0.494 to −0.234, *P*<0.001; Supplementary Fig. S3). This suggests greater cognitive vulnerability to nociplastic pain in males with chronic pain.

### Longitudinal stability of executive function

Longitudinal CFA indicated partial scalar invariance over time, supporting the stability of executive function measurement across assessments,^35^ though full invariance was not achieved (Supplementary Table S11 and Supplementary Fig. S4).

### Longitudinal association between nociplastic pain severity and executive function

**Fig 3:**
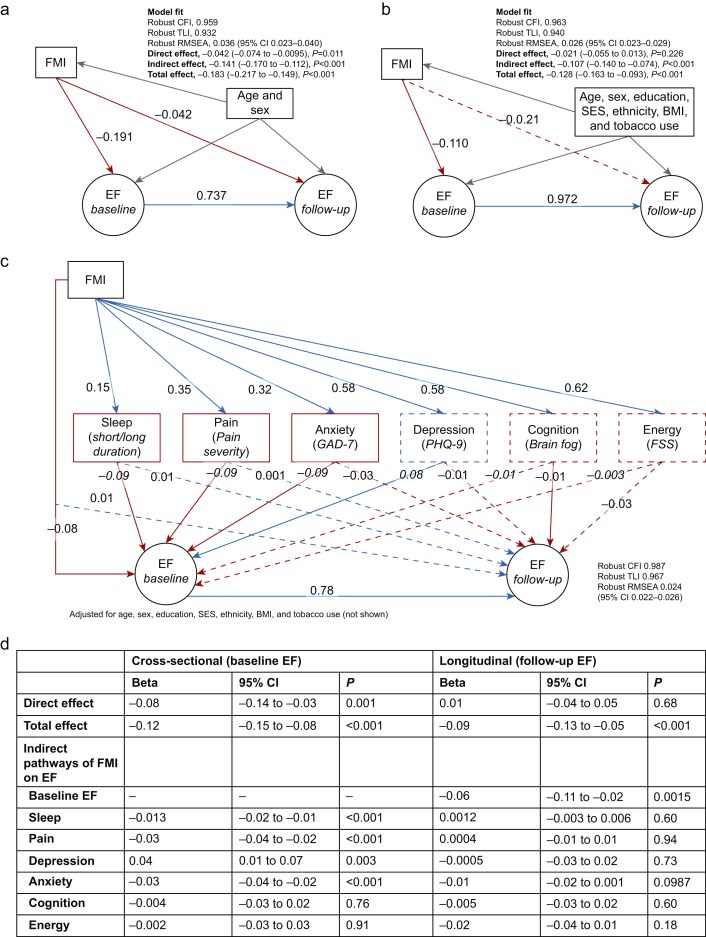
Structural equation models of the relationship between the fibromyalgia index and executive function. (a and b) No significant longitudinal association between nociplastic pain severity and decline in executive function. Structural equation models illustrating the relationship between fibromyalgia index (FMI) and executive function (EF) at follow-up, mediated by baseline EF. Blue lines indicate positive associations, red lines indicate negative associations, solid lines denote significant paths, and dashed lines denote non-significant paths. Panel a adjusts for age and sex, while panel b includes additional adjustments for education, socioeconomic status (SES), ethnicity, body mass index (BMI), and tobacco use. (a and d) Sleep, pain, and anxiety mediate the cross-sectional association between FMI and EF. Panel c depicts indirect pathways linking FMI to baseline EF via sleep duration, pain severity, anxiety, depression, cognitive complaints, and fatigue. Panel d summarises direct, indirect, and total effects. Model adjusted for age, sex, education, SES, ethnicity, BMI, and tobacco use, which are not shown for legibility. Covariances between the mediators were also modelled, which again are not depicted for clarity. Bootstrapped confidence intervals estimated with 5000 iterations. Model fit indices: robust comparative fit index (CFI), robust Tucker-Lewis index (TLI), and robust root mean square error of approximation (RMSEA). CI, confidence interval; FSS, fatigue severity scale.

In an SEM model adjusted for age and sex, FMI had a small negative direct effect on follow-up executive function (β −0.042, 95% CI −0.074 to −0.01, *P*=0.011; Fig. 3a). However, after full adjustment for confounders, this association was attenuated and non-significant (β −0.021, 95% CI −0.055 to 0.013, *P*=0.226; Fig. 3b). The indirect effect of FMI through baseline executive function remained significant (β −0.107, 95% CI −0.140 to −0.074, *P*<0.001), suggesting that the effect of nociplastic pain on cognition was primarily cross-sectional rather than associated with rate of cognitive decline. Detailed results are given in the Supplementary material (Appendix D and Supplementary Table S12).

### Mediation analysis

SPACE symptoms (Fig. 3c and d): higher FMI was associated with worse executive function at baseline through indirect effects involving short/long sleep duration (β −0.013; 95% CI −0.02 to −0.01; *P*<0.001), pain severity (β −0.03; 95% CI −0.04 to −0.02; *P*<0.001), and anxiety (β −0.03; 95% CI −0.04 to −0.02; *P*<0.001). However, no significant indirect effects were observed for follow-up executive function, suggesting these symptoms do not mediate cognitive decline.

**Fig 4:**
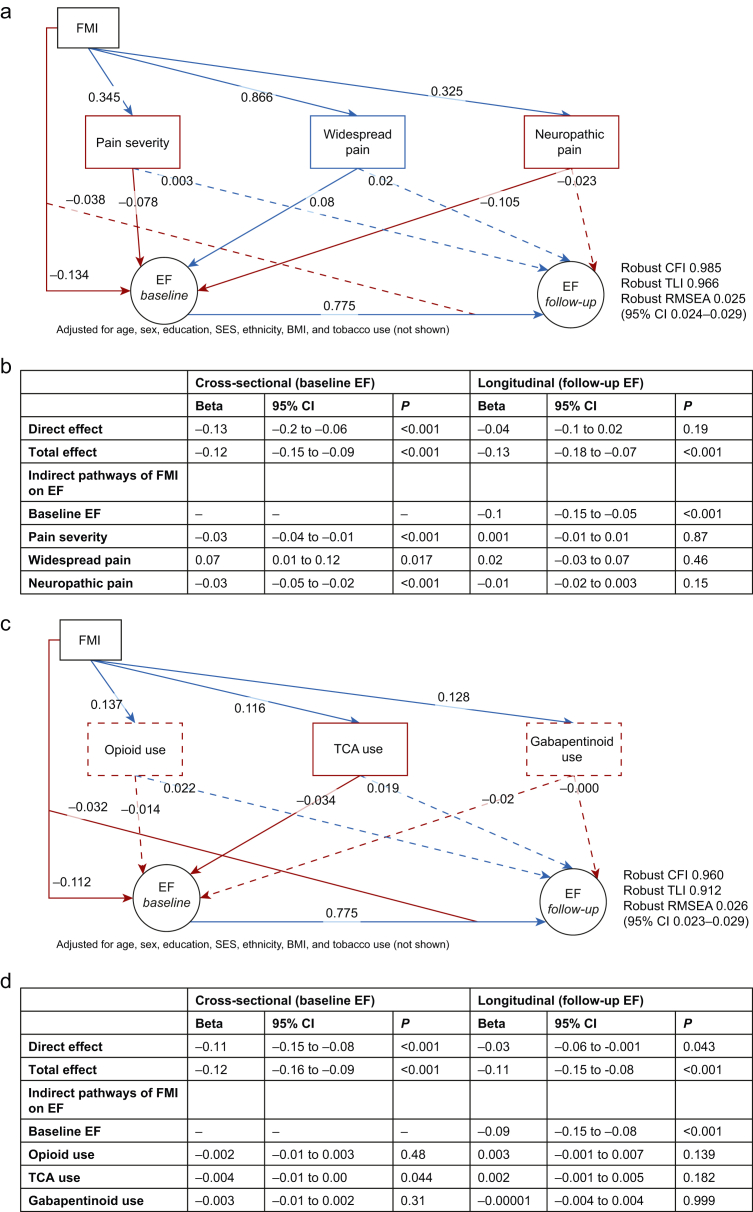
Structural equation models examining the relationship between fibromyalgia index, pain characteristics, analgesia use, and executive function. (a and b) Pain severity and neuropathic pain partially mediate the cross-sectional but not longitudinal relationship between nociplastic pain severity and executive function. Structural equation models illustrating the indirect effects of pain characteristics on the association between fibromyalgia index (FMI) and executive function (EF) at baseline and follow-up. Blue lines indicate positive associations, red lines indicate negative associations, solid lines denote significant paths, and dashed lines denote non-significant paths. Panel a shows associations with pain severity, widespread pain, and neuropathic pain, while panel b summarises direct, indirect, and total effects. (c and d) Analgesia use does not mediate the relationship between nociplastic pain severity and executive function. Panel c presents a model assessing the indirect pathways of FMI on EF through opioid, tricyclic antidepressant (TCA), and gabapentinoid use. Panel d summarises statistical estimates of direct and indirect effects. Models adjust for age, sex, education, socioeconomic status (SES), ethnicity, body mass index (BMI), and tobacco use. Covariances between mediators were included in the model but are omitted for clarity. Bootstrapped confidence intervals were estimated with 5000 iterations. Model fit indices: robust comparative fit index (CFI), robust Tucker-Lewis index (TLI), and robust root mean square error of approximation (RMSEA). CI, confidence interval.

Pain characteristics (Fig. 4a and b): at baseline, pain severity (β −0.03; 95% CI −0.04 to −0.01; *P*<0.001), and neuropathic pain features (β −0.03; 95% CI −0.05 to −0.02; *P*<0.001) partially mediated the cross-sectional association between FMI and executive function. However, as with the SPACE symptoms, no significant indirect effects were observed for follow-up executive function.

Analgesic use (Fig. 4c and d): no significant mediation effects were found for opioid or gabapentinoid use. A marginal indirect effect was observed for TCA use (β −0.004; 95% CI −0.01 to 0.00; *P*=0.044), but its clinical significance is unclear.

Detailed results for mediation analyses are given in the Supplementary material (Appendix E and Supplementary Tables S13–S15).

### Sensitivity analyses

Findings were robust across the four sensitivity analyses. The relationship between nociplastic pain severity and cognitive function was observed across all three tasks. Results remained unchanged when stratified by the timing of assessments, including pre- *vs* post-COVID-19 periods (for detailed results, see Supplementary material [Appendix F, Supplementary Figs S5–S7, and Supplementary Tables S16–S18]).

## Discussion

This study is the first large-scale, longitudinal investigation into the association between nociplastic pain and executive function. We show that nociplastic pain severity is associated with worse executive function cross-sectionally, particularly in males. However, reassuringly, it does not predict cognitive decline over time, addressing an important gap in the literature. Mediation analyses reveal that abnormal sleep duration, anxiety, pain intensity, and neuropathic pain features partially explain the cross-sectional relationship, identifying potential targets for intervention.

### Existing literature

While chronic pain has been linked to memory decline and dementia,^2^ evidence for an association with executive function decline is mixed. This question is important, as executive dysfunction can affect daily functioning, decision-making, and quality of life. Studies using the English Longitudinal Study of Ageing cohort have linked chronic pain and pain interference to cognitive impairment in older adults,^43^ while others have found no such association.^7^ Prior UK Biobank studies have linked multisite chronic pain to dementia risk but have not examined nociplastic pain severity or longitudinal cognitive trajectories.^44^ This study builds on existing work by using the FMI to assess nociplastic pain severity, an approach not previously applied in large population-based cohorts.

#### Sex differences

Although nociplastic pain is more prevalent in females, its association with executive dysfunction was stronger in males. Preclinical models suggest males may be more vulnerable to pain-related cognitive impairments because of differences in prefrontal plasticity.^20^ A neuroimaging study by Linnman and colleagues^21^ found that males exhibit stronger pain-induced connectivity between emotion- and cognition-related brain regions, such as the amygdala, prefrontal cortex, and periaqueductal grey, suggesting greater integration of affective and executive control circuits during pain processing, potentially contributing to increased vulnerability to pain-related cognitive disruption in males.

### Mediation analysis

Although there was no longitudinal association, the cross-sectional association between nociplastic pain severity and executive function was partially mediated by abnormal sleep duration, anxiety, pain intensity, and neuropathic symptoms, suggesting potential therapeutic targets.

#### Sleep

Both insufficient and excessive sleep duration are linked to worse cognition.^14^ Non-restorative sleep, a hallmark of chronic pain, may impair executive function. Our findings relate specifically to abnormal sleep duration, rather than subjective sleep disturbance. Short sleep may reflect insufficient restorative processes, while long sleep may reflect fragmented or poor-quality sleep.^45^^,^^46^ These findings align with the hyperarousal theory of insomnia, which posits that disrupted brain network modulation leads to distractibility and cognitive inefficiency.^47^ Sleep disturbances are common in chronic pain and correlate with self-reported cognitive difficulties in fibromyalgia.^48^ Our findings suggest that sleep duration may be a potential modifiable factor linking nociplastic pain and cognitive dysfunction.

#### Pain characteristics

Pain intensity and neuropathic features were associated with worse executive function, potentially through attentional interference. High-intensity pain disrupts cognitive resources, particularly on tasks requiring cognitive flexibility.^49^ Meanwhile, neuropathic pain questionnaires, such as the DN4 or PainDETECT, may capture features of central sensitisation, including burning, tingling, and electric shock-like sensations, that are common in nociplastic conditions. Although originally developed to screen for neuropathic pain, these tools may reflect centrally-mediated mechanisms that contribute to cognitive dysfunction, particularly in the absence of clear peripheral nerve injury.^50^ Furthermore, in one recent study, neuropathic pain patients perform worse on sustained attention tasks compared with fibromyalgia patients, despite overlapping symptom profiles.^51^

Interestingly, after controlling for pain severity and neuropathic features, widespread pain was positively associated with executive function. This contradicts prior research suggesting that widespread pain is associated with worse cognition.^52^ Widespread pain may serve as a surrogate marker of pain severity, but its impact on cognition is driven by pain intensity and neuropathic-like features rather than distribution alone.

#### Anxiety and depression

Anxiety significantly mediated the cross-sectional pain–executive function relationship, consistent with prior work linking anxiety to cognitive rigidity and impaired attention shifting.^53^ Anxiety and chronic pain share neurobiological characteristics, such as heightened amygdala activation.^54^ Anxiety can reinforce chronic pain through hypervigilance and increased pain sensitivity, creating a feedback loop of cognitive-affective dysfunction.

Conversely, depressive symptoms did not mediate the association, which may reflect the psychological profile of nociplastic pain conditions such as fibromyalgia, where anxiety is often more prominent than depression.^55^ While both are common, anxiety may have a stronger influence as a result of heightened sensory sensitivity and hypervigilance. These features align with neurocognitive mechanisms such as increased amygdala responsivity,^54^ potentially explaining why only anxiety significantly mediated the pain–executive function relationship.

#### Brain fog and fatigue

Neither brain fog nor fatigue significantly mediated the pain–executive function relationship in this study. Brain fog may reflect general cognitive inefficiency rather than specific executive dysfunction, while fatigue may primarily affect motivation and task persistence.^56^

#### Analgesia use

We found no significant mediation effect of opioid use, aligning with recent systematic reviews indicating no clear cognitive decline associated with chronic opioid therapy.^16^^,^^17^ However, self-reported analgesia use may be subject to recall bias, and the inability to account for dosage or duration of use remains a limitation.

### Strengths and limitations

Strengths include the large sample size, longitudinal design, and use of a latent executive function measure to reduce measurement error and account for factorial invariance over time. Limitations include the relatively short follow-up period, which precludes conclusions about long-term trajectories in executive function. Reverse causation remains a possibility, as cognitive function may influence pain perception and reporting. Reliance on self-reported measures introduces recall bias, particularly for sleep and analgesia use. Additionally, the sample may not fully represent individuals with high-impact chronic pain or cognitive impairment who are less likely to participate in research. UK Biobank’s healthy volunteer bias may also limit generalisability. Finally, while adjustments for confounders were made, unmeasured factors such as childhood trauma or other early life experiences may contribute to the observed associations. We also did not explicitly model the interaction between tricyclic antidepressant use and depressive symptoms, as the number of users was small and insufficient for robust stratified analysis.

### Conclusions

While nociplastic pain is cross-sectionally associated with executive dysfunction, it does not predict cognitive decline over time—an important and reassuring distinction. Abnormal sleep duration and anxiety partially mediate this relationship, highlighting them as key therapeutic targets for improving cognitive performance in chronic pain patients. Future research should focus on long-term pain–cognition interactions and evaluate whether interventions targeting pain, sleep, and anxiety can mitigate cognitive deficits in nociplastic pain.

## Authors’ contributions

Conceived and designed the analysis, collected the data, contributed data or analysis tools, performed the analysis, wrote the manuscript, directly accessed and verified the data: EK, AI

Contributed by conceiving and designing the analysis and writing the manuscript: XYT, AS, TRM, TN, IT

Approved the final version of the manuscript: all authors

## Data sharing

Data are available upon application to UK Biobank. All analysis codes will be made publicly available upon publication on an online repository.

## Funding

The National Institute for Health Research (NIHR) Biomedical Research Centre, Oxford (NIHR203316); EK was supported by an NIHR Pfizer Doctoral Fellowship for this research project (NIHR301808). The Centre for Integrative Neuroimaging was supported by core funding from the Wellcome Trust (203139/Z/16/Z, 203139/A/16/Z). This paper presents independent research funded by the NIHR and Pfizer. The views expressed are those of the author(s) and not necessarily those of the NHS, the NIHR, the Department of Health and Social Care or Pfizer.

## Declaration of interest

EK was supported by a National Institute for Health Research (NIHR) Pfizer Doctoral Fellowship for this research project (NIHR301808). All other authors declare that they have no conflicts of interest.

## References

[1] Gilbert S.J., Burgess P.W. (2008). Executive function. Curr Biol.

[2] Whitlock E.L., Diaz-Ramirez L.G., Glymour M.M., Boscardin W.J., Covinsky K.E., Smith A.K. (2017). Association between persistent pain and memory decline and dementia in a longitudinal cohort of elders. JAMA Intern Med.

[3] Berryman C., Stanton T.R., Bowering K.J., Tabor A., McFarlane A., Moseley G.L. (2014). Do people with chronic pain have impaired executive function? A meta-analytical review. Clin Psychol Rev.

[4] Berryman C., Stanton T.R., Jane Bowering K., Tabor A., McFarlane A., Lorimer Moseley G. (2013). Evidence for working memory deficits in chronic pain: a systematic review and meta-analysis. Pain.

[5] Bell T., Trost Z., Buelow M.T. (2018). Meta-analysis of cognitive performance in fibromyalgia. J Clin Exp Neuropsychol.

[6] van der Leeuw G., Ayers E., Leveille S.G., Blankenstein A.H., van der Horst H.E., Verghese J. (2018). The effect of pain on major cognitive impairment in older adults. J Pain.

[7] Veronese N., Koyanagi A., Solmi M. (2018). Pain is not associated with cognitive decline in older adults: a four-year longitudinal study. Maturitas.

[8] Rouch I., Dorey J.-M., Strippoli M.-P.F. (2021). Does cognitive functioning predict chronic pain in older adult? Results from the CoLaus|PsyCoLaus longitudinal study. J Pain.

[9] Raja S.N., Carr D.B., Cohen M. (2020). The revised International Association for the Study of Pain definition of pain: concepts, challenges, and compromises. Pain.

[10] Schrepf A., Williams D.A., Gallop R. (2018). Sensory sensitivity and symptom severity represent unique dimensions of chronic pain: a MAPP Research Network study. Pain.

[11] Jung C.M., Ronda J.M., Czeisler C.A., Wright Jr KP. (2011). Comparison of sustained attention assessed by auditory and visual psychomotor vigilance tasks prior to and during sleep deprivation. J Sleep Res.

[12] Drummond S.P., Anderson D.E., Straus L.D., Vogel E.K., Perez V.B. (2012). The effects of two types of sleep deprivation on visual working memory capacity and filtering efficiency. PloS One.

[13] Moldofsky H., Scarisbrick P., England R., Smythe H. (1975). Musculosketal symptoms and non-REM sleep disturbance in patients with “fibrositis syndrome” and healthy subjects. Psychosom Med.

[14] Tai X.Y., Chen C., Manohar S., Husain M. (2022). Impact of sleep duration on executive function and brain structure. Commun Biol.

[15] Veldhuijzen D.S., Kenemans J.L., de Bruin C.M., Olivier B., Volkerts E.R. (2006). Pain and attention: attentional disruption or distraction?. J Pain.

[16] Pask S., Dell'Olio M., Murtagh F.E.M., Boland J.W. (2020). The effects of opioids on cognition in older adults with cancer and chronic noncancer pain: a systematic review. J Pain Symptom Manage.

[17] Kendall S.E., Sjøgren P., Pimenta C.A.M., Højsted J., Kurita G.P. (2010). The cognitive effects of opioids in chronic non-cancer pain. Pain.

[18] Oh G., Moga D.C., Fardo D.W., Abner E.L. (2022). The association of gabapentin use and change of cognitive and functional status in older adults with normal cognition. Front Pharmacol.

[19] Pickering G., Pereira B., Clere F. (2014). Cognitive function in older patients with postherpetic neuralgia. Pain Pract.

[20] Shiers S., Pradhan G., Mwirigi J. (2018). Neuropathic pain creates an enduring prefrontal cortex dysfunction corrected by the type II diabetic drug metformin but not by gabapentin. J Neurosci.

[21] Linnman C., Beucke J.-C., Jensen K.B., Gollub R.L., Kong J. (2012). Sex similarities and differences in pain-related periaqueductal gray connectivity. Pain.

[22] Allen N.E., Sudlow C., Peakman T., Collins R. (2014). UK biobank data: come and get it. Sci Transl Med.

[23] Wolfe F., Clauw D.J., Fitzcharles M.A. (2016). Revisions to the 2010/2011 fibromyalgia diagnostic criteria. Semin Arthritis Rheum.

[24] Brummett C.M., Janda A.M., Schueller C.M. (2013). Survey criteria for fibromyalgia independently predict increased postoperative opioid consumption after lower-extremity joint arthroplasty: a prospective, observational cohort study. Anesthesiology.

[25] For ref 25: UK Biobank. Category 100026: Cognitive function [Internet]. Oxford: UK Biobank; [cited 2025 Jul]. Available from: https://biobank.ndph.ox.ac.uk/showcase/label.cgi?id=100026ref

[26] Fawns-Ritchie C., Deary I.J. (2020). Reliability and validity of the UK Biobank cognitive tests. PLoS One.

[27] Kaplan C.M., Kelleher E., Irani A., Schrepf A., Clauw D.J., Harte S.E. (2024). Deciphering nociplastic pain: clinical features, risk factors and potential mechanisms. Nat Rev Neurol.

[28] Townsend P. (1987). Deprivation. J Soc Policy.

[29] Watson N.F., Badr M.S., Belenky G. (2015). Recommended amount of sleep for a healthy adult: a joint consensus statement of the American Academy of Sleep Medicine and Sleep Research Society. Sleep.

[30] UK Biobank. Category 100075: Verbal interview medication [Internet]. Oxford: UK Biobank; [cited 2025 Jul]. Available from: https://biobank.ndph.ox.ac.uk/showcase/label.cgi?id=100075

[31] Little T.D. (2013).

[32] Bollen K.A. (2014).

[33] Lyall D.M., Cullen B., Allerhand M. (2016). Cognitive test scores in UK Biobank: data reduction in 480,416 participants and longitudinal stability in 20,346 participants. PLoS One.

[34] Crawford J.R., Garthwaite P.H. (2009). Percentiles please: the case for expressing neuropsychological test scores and accompanying confidence limits as percentile ranks. Clin Neuropsychol.

[35] Widaman K.F., Ferrer E., Conger R.D. (2010). Factorial invariance within longitudinal structural equation models: measuring the same construct across time. Child Dev Perspect.

[36] Cheung G.W., Rensvold R.B. (2002). Evaluating goodness-of-fit indexes for testing measurement invariance. Struct Equ Modeling.

[37] Chen F.F. (2007). Sensitivity of goodness of fit indexes to lack of measurement invariance. Struct Equ Modeling.

[38] Rutkowski L., Svetina D. (2014). Assessing the hypothesis of measurement invariance in the context of large-scale international surveys. Educ Psychol Meas.

[39] Wicherts JM. (2016 Oct). The importance of measurement invariance in neurocognitive ability testing. Clin Neuropsychol.

[40] Baron R.M., Kenny D.A. (1986). The moderator–mediator variable distinction in social psychological research: conceptual, strategic, and statistical considerations. J Pers Soc Psychol.

[41] Little T.D., Card N.A., Bovaird J.A., Preacher K.J., Crandall C.S. (2007). Structural equation modeling of mediation and moderation with contextual factors. Modeling Contextual Effects in Longitudinal Studies.

[42] Cheung G.W., Lau R.S. (2008). Testing mediation and suppression effects of latent variables: bootstrapping with structural equation models. Organ Res Methods.

[43] Rong W., Zhang C., Zheng F., Xiao S., Yang Z., Xie W. (2021). Persistent moderate to severe pain and long-term cognitive decline. Eur J Pain.

[44] Zhao W., Zhao L., Chang X., Lu X., Tu Y. (2023). Elevated dementia risk, cognitive decline, and hippocampal atrophy in multisite chronic pain. Proc Natl Acad Sci U S A.

[45] Bédard M.A., Montplaisir J., Richer F., Rouleau I., Malo J. (1991). Obstructive sleep apnea syndrome: pathogenesis of neuropsychological deficits. J Clin Exp Neuropsychol.

[46] Bjurstrom M.F., Irwin M.R. (2016). Polysomnographic characteristics in nonmalignant chronic pain populations: A review of controlled studies. Sleep Med Rev.

[47] Riemann D., Spiegelhalder K., Feige B. (2010). The hyperarousal model of insomnia: a review of the concept and its evidence. Sleep Med Rev.

[48] Whibley D., Williams D., Clauw D., Kratz A. (2019). The association between non-restorative sleep and diurnal patterns of cognitive function and fatigue in people with fibromyalgia and matched controls. Arthritis Rheumatol.

[49] Seminowicz D.A., Davis K.D. (2007). Interactions of pain intensity and cognitive load: the brain stays on task. Cereb Cortex.

[50] Koroschetz J., Rehm S.E., Gockel U. (2011). Fibromyalgia and neuropathic pain--differences and similarities. A comparison of 3057 patients with diabetic painful neuropathy and fibromyalgia. BMC Neurol.

[51] Jacobsen H.B., Stiles T.C., Stubhaug A., Landrø N.I., Hansson P. (2021). Comparing objective cognitive impairments in patients with peripheral neuropathic pain or fibromyalgia. Sci Rep.

[52] Lee D.M., Pendleton N., Tajar A. (2010). Chronic widespread pain is associated with slower cognitive processing speed in middle-aged and older European men. Pain.

[53] Warren S.L., Heller W., Miller G.A. (2021). The structure of executive dysfunction in depression and anxiety. J Affect Disord.

[54] Zhuo M. (2016). Neural mechanisms underlying anxiety-chronic pain interactions. Trends Neurosci.

[55] McWilliams L.A., Cox B.J., Enns M.W. (2003). Mood and anxiety disorders associated with chronic pain: an examination in a nationally representative sample. Pain.

[56] Teodoro T., Edwards M.J., Isaacs J.D. (2018). A unifying theory for cognitive abnormalities in functional neurological disorders, fibromyalgia and chronic fatigue syndrome: systematic review. J Neurol Neurosurg Psychiatry.

