# Supplementary material for: Association of nociplastic pain with executive function decline in a longitudinal cohort of middle-age adults: a prospective cohort study
Source: Br J Anaesth. 2025 Aug 20;135(6):1717–29. doi: 10.1016/j.bja.2025.08.002 (PMC12799397; doi:10.1016/j.bja.2025.08.002)
Supplement: Multimedia component 1 [file mmc1.docx]

Table of Contents

[Appendix A: Data preparation 6](#_Toc196337516)

[Appendix B: Longitudinal stability of executive function 7](#_Toc196337517)

[Appendix C: Cross-sectional analysis: detailed regression results & diagnostics 18](#_Toc196337518)

[Appendix D: Longitudinal CFA 27](#_Toc196337519)

[Appendix E. Mediation analyses 28](#_Toc196337520)

[Appendix F. Sensitivity analyses 32](#_Toc196337521)

[Individual cognitive tests 32](#_Toc196337522)

[Effect modification by sex 36](#_Toc196337523)

[Assessment time discrepancies & stratification by COVID-19 period 38](#_Toc196337524)

[References 40](#_Toc196337525)

[**Figure S1. Flow diagram of UK Biobank assessments** 5](#_Toc196337526)

[**Figure S2. Path diagrams for latent variables for executive function at baseline** 17](#_Toc196337527)

[**Figure S3. Sex Differences in the Association Between Fibromyalgia Index (FMI) and Executive Function (EF)** 21](#_Toc196337528)

[**Figure S4. Partial scalar invariance exists over time for executive function for UK Biobank participants with chronic time.** 26](#_Toc196337529)

[**Figure S5. Higher fibromyalgia index (FMI) associated with worse performance on all three tests of executive function in adults with chronic pain.** 32](#_Toc196337530)

[**Figure S6. Cross-sectional association between fibromyalgia index (FMI) and cognitive test performance by sex among participants with chronic pain.** 37](#_Toc196337531)

[**Figure S7. Timing of cognitive assessments in relation to pain assessment and COVID-19 pandemic does not change interpretation of results.** 39](#_Toc196337532)

[**Table S1. Field IDs of variables from UK Biobank used in analysis** 8](#_Toc196337533)

[**Table S2. Codes used to derive self-reported analgesia use from questionnaire at time of first imaging visit**. 10](#_Toc196337534)

[**Table S3. Baseline characteristics of participants included in longitudinal analysis of FMI and executive function.** 12](#_Toc196337535)

[**Table S4. Comparison of baseline characteristics of participants in cross-sectional analysis who also had follow-up cognitive outcome data available.** 14](#_Toc196337536)

[**Table S5. Cognitive test scores at baseline and follow-up for participants with and without chronic pain. All results adjusted for age at the time of the test**. 16](#_Toc196337537)

[**Table S6. Cross-sectional relationship between fibromyalgia index (FMI) score and executive function, with chronic pain interaction.** 19](#_Toc196337538)

[**Table S7. Cross-sectional relationship between fibromyalgia index (FMI) score and executive function rank, with chronic pain interaction.** 20](#_Toc196337539)

[**Table S8. Cross-sectional relationship between fibromyalgia index (FMI) score and executive function, with sex interaction.** 22](#_Toc196337540)

[**Table S9. Cross-sectional relationship between fibromyalgia index (FMI) score and executive function, with sex interaction.** 23](#_Toc196337541)

[**Table S10. No evidence of collinearity present in model.** 24](#_Toc196337542)

[**Table S11. Model Fit Indices for Factorial Invariance Testing of Executive Function Across Time Points.** 25](#_Toc196337543)

[**Table S12. Longitudinal SEM analysis of the indirect and direct effects of nociplastic pain severity (FMI) on executive function.** 27](#_Toc196337544)

[**Table S13. Mediation analysis of the indirect and direct effects of SPACE symptom domains on the association between Fibromyalgia Index (FMI) score and executive function.** 28](#_Toc196337545)

[**Table S14. Mediation analysis of the indirect and direct effects of pain characteristics on the association between Fibromyalgia Index (FMI) score and executive function.** 30](#_Toc196337546)

[**Table S15. Mediation analysis of the indirect and direct effects of self-reported analgesia use on the association between Fibromyalgia Index (FMI) score and executive function.** 31](#_Toc196337547)

[**Table S16. Cross-sectional relationship between fibromyalgia index (FMI) score and Matrix Pattern test score, with interaction with chronic pain.** 33](#_Toc196337548)

[**Table S17. Cross-sectional relationship between fibromyalgia index (FMI) score and Trail Making Test score, with interaction with chronic pain.** 34](#_Toc196337549)

[**Table S18. Cross-sectional relationship between fibromyalgia index (FMI) score and Digit-Symbol Substitution Test score, with interaction with chronic pain.** 35](#_Toc196337550)


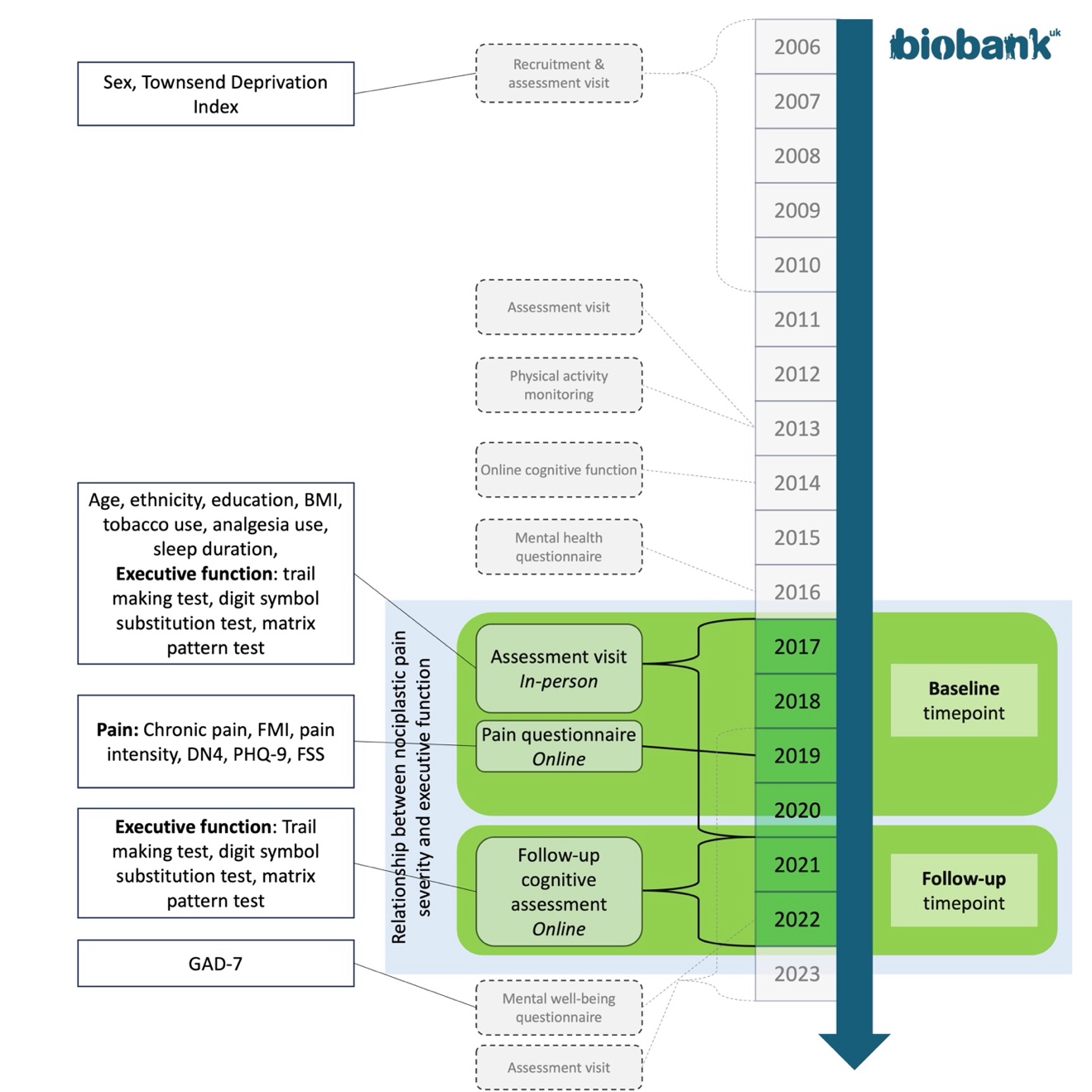


**Figure S1. Flow diagram of UK Biobank assessments**

A flow diagram of the timeline for the main assessments in UK Biobank. The assessments used in the current study are highlighted in green. All participants for whom UK Biobank had a current email address (~333,000) were invited to attend follow-up visits and complete online questionnaires. Commencing 2020, UK Biobank began sending postal invitations for the follow-up imaging visit. A small number (<0.5%) of participants have withdrawn or moved outside the UK. An expanded battery of cognitive assessments was introduced in imaging visits conducted after December 2016, and participants who attended the imaging visit prior to this were not included. The cognitive assessment undertaken during the first imaging visit between 2017 and 2020 was taken was the baseline timepoint for this study. The online pain questionnaire taken in 2019 was taken as the baseline assessment for pain. The online cognitive assessment performed in 2021-2022 was the follow-up assessment for this study. Sex and Townsend deprivation index were only assessed at recruitment. As no validated measure of anxiety was assessed at the time of the imaging visit or pain questionnaire, the GAD-7 from the 2022 mental well-being questionnaire was used. For the cross-sectional analysis, participants who completed cognitive tests at the first imaging visit and the experience of pain questionnaire were included. For the longitudinal analysis, participants who subsequently also completed the follow-up online cognitive assessment were included. BMI, body mass index. FMI, fibromyalgia index. DN4, douleur neuropathique 4. PHQ-9, patient health questionnaire 9-item. FSS, fatigue severity scale. GAD-7, general anxiety disorder 7-item.

# Appendix A: Data preparation

Outliers in the trail making test (>|3| SD) were winsorised by capping values within the observed range. This approach reduced the influence of extreme values, which may reflect measurement error or rare but valid individual differences, on model specification.

Given the strong confounding influence of age on cognitive performance, which was not of primary interest in the present study, the individual cognitive tests were adjusted for age prior to CFA.^1^ To account for the varying impact of age across tests, polynomial regression was used to model the relationship between age and each test. Polynomial fits of increasing degrees were compared using likelihood ratio tests to identify the model which best captures the relationship with age. At baseline, quadratic models fit the SDS and matrix pattern tests, while a cubic model fit the TMT; at follow-up, a linear model fit the SDS test, a quadratic model fit the TMT, and a cubic model fit the matrix pattern test. Residuals from these models, representing variance in test scores independent of age, were extracted. This adjustment effectively removed age-related variance, as confirmed by near-zero coefficients and nonsignificant associations with age in the adjusted models.

The variables were then re-scaled using the proportion of maximum scaling (POMS) method to give a common scale of 0 to 1, to reduce model misspecification which may occur when variables on different scales are included in factor analysis^2^. TMT scores were reverse coded so that higher scores indicated better cognitive performance across all tests.

To aid interpretation and communication of results, the cognitive test outcomes and latent factor for executive function were also transformed into centile ranks, where the 1^st^ centile is the worst performer, and the 100^th^ centile is the best performer.^3^

# Appendix B: Longitudinal stability of executive function

To assess the factorial invariance and longitudinal stability of executive function over time, longitudinal CFA was conducted using the three cognitive tests described in methods section. A two-factor, three-item CFA model was constructed,^2^ with residual covariances estimated between the same tasks across time.

Measurement invariance was assessed by fitting four CFA models with progressively stricter constraints:

1. **Configural invariance**: Allowed freely estimated factor loadings, intercepts, and item variances across both time points.
2. **Weak (metric) invariance**: Constrained factor loadings to be equal across time, while intercepts and variances were freely estimated.
3. **Partial strong (scalar) invariance**: Constrained factor loadings of all variables, and intercepts of SDS and matrix pattern test to be equal across both time points, allowing the TMT intercept to vary.
4. **Strong (scalar) invariance**: Constrained all factor loadings and intercepts to be equal across time, allowing only item variances to vary.

Models were fitted using robust maximum likelihood estimation. Model fit was assessed using the robust CFI, TLI, RMSEA, and Standardised Root Mean Square Residual (SRMR). Incremental changes in fit indices were used to determine invariance between models^4^. A threshold of ≤0.01 for ΔCFI and ΔTLI,^5^ ≤0.015 for ΔRMSEA,^6^ and ≤0.03 for ΔSRMR^7^ was used to assess invariance between models.

**Table S1. Field IDs of variables from UK Biobank used in analysis**

| **Variable** | **UKB Datafield** | **Instance** | **Comments** |
| --- | --- | --- | --- |
| Age | 21003 | 2 |  |
| Sex | 31 | 0 |  |
| Ethnicity | 21000 | 2 | Binarised to white or non-white. Imputed with instance 0 if missing at 2 |
| Townsend Deprivation Index | 22189 | 0 |  |
| Education | 6138 | 2 | Binarised to University Degree and No Degree. Instance 0 or 10722 used if 6138 missing. |
| Employment status | 6142 | 2 | Binarised to employed or not employed |
| Smoking | 20116 | 2 | Binarised to current smoking or not current smoker |
| Alcohol use | 1558 | 2 | Binarised to current alcohol use or no current alcohol use |
| BMI | 21001 | 2 | Instance 0 or 23104 used if 21001 missing |
| Sleep duration | 1160 | 2 |  |
| Abnormal sleep duration | 1160 | 2 | Binarised to <7 & >9 or 7-9 |
| Opioid use | 20003 | 2 | See below for codes used to identify opioids |
| Tricyclic antidepressant use | 20003 | 2 | See below for codes used to identify TCAs |
| Gabapentinoid use | 20003 | 2 | See below for codes used to identify gabapentinoids |
| Exclusion | 20002 | 0 | If contained codes: 1262 (dementia), 1263 (Parkinson's), 1289 (psychosis), or 1291 (bipolar disorder) |
| Chronic pain | 120019 | PQ | Answered "Yes" |
| Widespread Pain Index | 120039 | PQ | Sum of the number of pain areas. Those who reported “Undisclosed area” were regarded as missing |
| Fatigue | 120040 | PQ |  |
| Unrefreshing sleep | 120041 | PQ |  |
| Cognitive difficulties | 120042 | PQ |  |
| Abdominal pain | 120043 | PQ |  |
| Depression | 120044 | PQ |  |
| Headache | 120045 | PQ |  |
| Symptom Severity Scale |  | PQ | sum of 120039-120045 |
| Fibromyalgia Index |  | PQ | Sum of Widespread Pain Index and Symptom Severity Scale |
| Pain severity (NRS) | 120022 & 12086 | PQ |  |
| Depression (PHQ-9) | 120104-120112 | PQ | Sum of 120104-120112 |
| Anxiety (GAD-7) | 29058-29064 | MWBQ | sum of 29058-29064 |
| Fatigue Severity Scale (FSS) | 120119-120127 | PQ | Sum of 120119-120127, not answered if 120018 is "No" and 120040 is "No problem". In that case the variable was set at the minimum value (i.e. 9) |
| Neuropathic Pain (DN4) | 120046-120052 | PQ | Sum of 120046-120052. Only answered participant reported chronic pain in a body site (not answered if participant reported chronic pain "all over the body" |
| **Cognitive outcomes** |  |  |  |
| **Trail making test at baseline** |  | **2** | **Difference between trail A & B** |
| Time taken for trail A | 6348 | 2 |  |
| Time taken for trail B | 6350 | 2 |  |
| **Digit symbol substitution test at baseline** | |  | **Proportion of attempts correct** |
| Number of attempts | 23323 | 2 |  |
| Number correct | 23324 | 2 |  |
| **Matrix Pattern test at baseline** |  |  | **Proportion of attempts correct** |
| Number of attempts | 6374 | 2 |  |
| Number correct | 7373 | 2 |  |
| **Trail making test at follow-up** |  |  | **Difference between trail A & B** |
| Time taken for trail A | 20156 | 1 |  |
| Time taken for trail B | 20157 | 1 |  |
| **Digit symbol substitution test at follow-up** | |  | **Proportion of attempts correct** |
| Number of attempts | 20195 | 1 |  |
| Number correct | 20159 | 1 |  |
| **Matrix Pattern test at follow-up** | |  | **Proportion of attempts correct** |
| Number of attempts | 20761 | 1 |  |
| Number correct | 20760 | 1 |  |
| Baseline date | 53 | 2 |  |
| Date of Pain Questionnaire | 120128 | PQ |  |
| Matrix Pattern Test date | 20765 | 1 |  |
| Symbol Digit Substitution test date | 20137 | 1 |  |
| Trail Making Test date | 20136 | 1 |  |
| Date of Follow-up Cognition |  |  | Average of 20765, 20137, and 20136 |
| Age at pain assessment |  |  | Derived from field 31 and date of Pain Questionaire |
| Follow-up time from baseline to pain questionnaire | |  | The difference between baseline date (53) and pain questionnaire date (120128) |
| Follow-up time from baseline to follow-up cognition | | | The difference between pain questionnaire date (120128) and follow-up cognition date |

**Table S2. Codes used to derive self-reported analgesia use from questionnaire at time of first imaging visit**.

All codes derived from field ID 20002 at instance 2 in UK Biobank.

| **Variable** | **Codes** | | |
| --- | --- | --- | --- |
| Opioid | 1141170964 | 1141170966 | 1141170972 |
|  | 1141171052 | 1141171054 | 1141171066 |
|  | 1140856422 | 1140856354 | 1140856356 |
|  | 1140882392 | 1140882268 | 1140862988 |
|  | 1140871402 | 1140884464 | 1140878030 |
|  | 1141190956 | 1140925778 | 1140868286 |
|  | 1140871906 | 1140871910 | 1140871902 |
|  | 1140871926 | 1140871920 | 1140871924 |
|  | 1141190656 | 1141165512 | 1141169692 |
|  | 1141192990 | 1141192992 | 1141192994 |
|  | 1140871726 | 1140871776 | 1140871730 |
|  | 1140871798 | 1140871728 | 1140871782 |
|  | 1140871684 | 1141151000 | 1141168650 |
|  | 1141189008 | 1141189064 | 1141189010 |
|  | 1140871682 | 1140928262 | 1140928266 |
|  | 1140880956 | 1141157470 | 1140879212 |
|  | 1141172878 | 1141172880 | 1141172882 |
|  | 1140856356 | 1140884388 | 1141100000 |
|  | 1141171038 | 1141171048 | 1141171050 |
|  | 1140856406 | 1140856416 | 1140856418 |
|  | 1140856442 | 1140856454 | 1140856456 |
|  | 1140884444 | 1140884452 | 1140865654 |
|  | 1140856406 | 1141175200 | 1140882394 |
|  | 1140856422 | 1140910402 | 1140871908 |
|  | 1140856456 | 1140856454 | 1140856458 |
|  | 1140884460 | 1140862910 | 1140855802 |
|  | 1141168794 | 1140864070 | 1140910376 |
|  | 1140871692 | 1140882116 | 1140882406 |
|  | 1140871708 | 1140864632 | 1140871796 |
|  | 1140871722 | 1140927388 | 1140927384 |
|  | 1140856340 | 1141189068 | 1141189066 |
|  | 1140856442 | 1141168648 | 1140871688 |
|  | 1140856214 | 1140923346 | 1140923344 |
|  | 1140880942 | 1141172936 | 1141172938 |
|  | 1140911836 | 1140911830 | 1140911832 |
|  | 1140856420 | 1140882114 | 1141167748 |
|  | 1140856458 | 1141192996 | 1141168122 |
|  | 1141187304 | 1140871786 | 1140923350 |
|  | 1140882396 | 1140871780 | 1141172876 |
|  | 1140871904 | 1140871686 | 1140911834 |
|  | 1140864536 |  |  |
|  |  |  |  |
| Tricylic antidepressants | 1140879616 | 1140867948 | 1140867938 |
|  | 1140867658 | 1140867818 | 1140867662 |
|  | 1140867668 | 1140867600 |  |
|  |  |  |  |
| Gabapentinoids | 1141200004 | 1140872228 | 1141200072 |
|  | 1140872236 |  |  |

**Table S3. Baseline characteristics of participants included in longitudinal analysis of FMI and executive function.**

Higher values of Townsend Deprivation Index indicate greater social deprivation. Nociplastic pain assessed using the fibromyalgia index (FMI), with higher scores indicating more severe nociplastic pain. The FMI is the sum of the widespread pain index (WPI) and symptom severity scale (SSS). Pain intensity measured using numeric rating scale (NRS) among participants who indicated they had chronic pain, higher values indicate more severe pain. Depression measured using Patient Health Questionnaire 9-item on the pain questionnaire, with higher scores indicating more severe depression symptoms. Anxiety measured using the General Anxiety Disorder 7-item on the Mental Health and Well-being questionnaire, with higher scores indicating more severe anxiety symptoms. Brain-fog measured using item on subjective cognitive difficulties on SSS, with higher scores indicating more severe brai- fog symptoms. Fatigue measured using the Fatigue Severity Scale (FSS) on the pain questionnaire, with higher scores indicating more severe fatigue symptoms. Note this questionnaire was only offered to participants who reported fatigue on the SSS. Neuropathic pain symptoms measured using the Doleur Neuropathique 4 (DN4) on the pain questionnaire, with higher scores indicating more severe neuropathic symptoms. SD, standard deviation.

|  | **Total** | **No Chronic pain** | **Chronic pain** |
| --- | --- | --- | --- |
|  | **(N=18898)** | **(N=8876)** | **(N=10022)** |
| Sex |  |  |  |
| Female | 9884 (52 %) | 4300 (48 %) | 5584 (56 %) |
| Male | 9014 (48 %) | 4576 (52 %) | 4438 (44 %) |
| Age (years) |  |  |  |
| Mean (SD) | 64.5 (7.35) | 64.5 (7.38) | 64.6 (7.33) |
| Townsend Deprivation Index |  |  |  |
| Mean (SD) | -1.91 (2.70) | -1.97 (2.67) | -1.85 (2.73) |
| Marital status |  |  |  |
| Married/Partner | 14301 (76 %) | 6791 (77 %) | 7510 (75 %) |
| Not married | 4534 (24 %) | 2063 (23 %) | 2471 (25 %) |
| Employment status |  |  |  |
| Employed | 7244 (38 %) | 3515 (40 %) | 3729 (37 %) |
| Retired | 10938 (58 %) | 5067 (57 %) | 5871 (59 %) |
| Unemployed/Other | 684 (4 %) | 282 (3 %) | 402 (4 %) |
| White ethnicity, % | 97.7% | 97.8% | 97.7% |
| University degree, % | 54.1% | 56.6% | 51.9% |
| Current tobacco use, % | 2.76% | 2.68% | 2.83% |
| Alcohol Use |  |  |  |
| Never | 1160 (6 %) | 520 (6 %) | 640 (6 %) |
| Rarely | 4007 (21 %) | 1771 (20 %) | 2236 (22 %) |
| Weekly | 10459 (55 %) | 5015 (57 %) | 5444 (54 %) |
| Daily | 3235 (17 %) | 1556 (18 %) | 1679 (17 %) |
| Body Mass Index (kg/m2) |  |  |  |
| Mean (SD) | 26.4 (4.43) | 25.9 (4.11) | 26.8 (4.66) |
| Fibromyalgia Index (0-31) |  |  |  |
| Mean (SD) | 3.52 (3.42) | 1.94 (1.91) | 4.93 (3.82) |
| Widespread Pain Index (0-19) |  |  |  |
| Mean (SD) | 1.24 (1.91) | 0.281 (0.767) | 2.09 (2.20) |
| Symptom Severity Scale (0-12) |  |  |  |
| Mean (SD) | 2.28 (2.07) | 1.66 (1.63) | 2.83 (2.26) |
| Sleep duration, self reported |  |  |  |
| Mean (SD) | 7.16 (1.00) | 7.22 (0.952) | 7.10 (1.04) |
| Sleep duration, self reported |  |  |  |
| <7 hours | 4341 (23 %) | 1762 (20 %) | 2579 (26 %) |
| 7-9 hours | 13240 (70 %) | 6507 (73 %) | 6733 (67 %) |
| >9 hours | 1243 (7 %) | 576 (6 %) | 667 (7 %) |
| Pain intensity, NRS (0-10) |  |  |  |
| Mean (SD) | 3.66 (2.57) | NA (NA) | 3.66 (2.57) |
| Depression (PHQ-9, 0-27) |  |  |  |
| Mean (SD) | 5.00 (6.79) | 3.14 (4.85) | 6.65 (7.76) |
| Anxiety (GAD-7, 0-21) |  |  |  |
| Mean (SD) | 1.80 (3.02) | 1.40 (2.66) | 2.15 (3.27) |
| Neuroticism, 0-12 |  |  |  |
| Mean (SD) | 3.25 (2.97) | 2.80 (2.80) | 3.64 (3.05) |
| Brain-fog (SSS) |  |  |  |
| Mean (SD) | 1.39 (0.570) | 1.30 (0.491) | 1.47 (0.622) |
| Fatigue Severity Scale (FSS) |  |  |  |
| Mean (SD) | 18.3 (13.0) | 15.2 (10.5) | 21.0 (14.3) |
| Doleur Neuropathique 4 (0-7) |  |  |  |
| Mean (SD) | 1.12 (1.36) | NA (NA) | 1.12 (1.36) |
| Opioid use | 1.12% | 0.15% | 1.98% |
| Tricyclic Antidepressant use | 1.30% | 0.53% | 2.14% |
| Gabapentinoid use | 0.85% | 0.12% | 1.49% |

**Table S4. Comparison of baseline characteristics of participants in cross-sectional analysis who also had follow-up cognitive outcome data available.**

UK Biobank participants in cross-sectional analysis who did not have follow-up cognitive outcome data compared to those with follow-up cognitive outcome data. Comparisons between groups were made using ANOVA for continuous variables, and chi-square test for categorical variables. SD, standard deviation. Higher values of Townsend Deprivation Index indicate greater social deprivation. Nociplastic pain assessed using the fibromyalgia index (FMI), with higher scores indicating more severe nociplastic pain. The FMI is the sum of the widespread pain index (WPI) and symptom severity scale (SSS). Pain intensity measured using numeric rating scale (NRS) among participants who indicated they had chronic pain, higher values indicate more severe pain. Depression measured using Patient Health Questionnaire 9-item on the pain questionnaire, with higher scores indicating more severe depression symptoms. Anxiety measured using the General Anxiety Disorder 7-item on the Mental Health and Well-being questionnaire, with higher scores indicating more severe anxiety symptoms. Brain-fog measured using item on subjective cognitive difficulties on SSS, with higher scores indicating more severe brain-fog symptoms. Fatigue measured using the Fatigue Severity Scale (FSS) on the pain questionnaire, with higher scores indicating more severe fatigue symptoms. Note this questionnaire was only offered to participants who reported fatigue on the SSS. Neuropathic pain symptoms measured using the Doleur Neuropathique 4 (DN4) on the pain questionnaire, with higher scores indicating more severe neuropathic symptoms. Executive function derived from a latent variable at baseline, as described in the text.

|  |  | **Included in longitudinal analysis** | |  |
| --- | --- | --- | --- | --- |
|  | **Total** | **No** | **Yes** | **P-value** |
|  | (N=35423) | (N=16525) | (N=18898) |  |
| Sex |  |  |  |  |
| Female | 18588 (52 %) | 8704 (53 %) | 9884 (52 %) | 0.796 |
| Male | 16835 (48 %) | 7821 (47 %) | 9014 (48 %) |  |
| Age (years) |  |  |  |  |
| Mean (SD) | 64.5 (7.38) | 64.4 (7.41) | 64.5 (7.35) | 0.136 |
| Townsend Deprivation Index |  |  |  |  |
| Mean (SD) | -1.90 (2.73) | -1.88 (2.76) | -1.91 (2.70) | 0.72 |
| Marital status |  |  |  |  |
| Married/Partner | 26540 (75 %) | 12239 (74 %) | 14301 (76 %) | 0.0045 |
| Not married | 8746 (25 %) | 4212 (25 %) | 4534 (24 %) |  |
| Employment status |  |  |  |  |
| Employed | 12567 (35 %) | 5323 (32 %) | 7244 (38 %) | <0.001 |
| Retired | 21544 (61 %) | 10606 (64 %) | 10938 (58 %) |  |
| Unemployed/Other | 1243 (4 %) | 559 (3 %) | 684 (4 %) |  |
| White ethnicity, % | 97.5% | 97.3% | 97.7% | 0.0287 |
| University degree, % | 52.9% | 51.6% | 54.1% | <0.001 |
| Current tobacco use, % | 2.78% | 2.80% | 2.76% | 0.982 |
| Alcohol Use |  |  |  |  |
| Never | 2305 (7 %) | 1145 (7 %) | 1160 (6 %) | 0.061 |
| Rarely | 7599 (21 %) | 3592 (22 %) | 4007 (21 %) |  |
| Weekly | 19409 (55 %) | 8950 (54 %) | 10459 (55 %) |  |
| Daily | 6033 (17 %) | 2798 (17 %) | 3235 (17 %) |  |
| Body Mass Index (kg/m2) |  |  |  |  |
| Mean (SD) | 26.4 (4.46) | 26.4 (4.50) | 26.4 (4.43) | 0.407 |
| Fibromyalgia Index (0-31) |  |  |  |  |
| Mean (SD) | 3.59 (3.49) | 3.67 (3.57) | 3.52 (3.42) | <0.001 |
| Widespread Pain Index (0-19) |  |  |  |  |
| Mean (SD) | 1.27 (1.97) | 1.30 (2.03) | 1.24 (1.91) | 0.0195 |
| Symptom Severity Scale (0-12) |  |  |  |  |
| Mean (SD) | 2.32 (2.09) | 2.37 (2.11) | 2.28 (2.07) | <0.001 |
| Sleep duration, self reported |  |  |  |  |
| Mean (SD) | 7.16 (1.02) | 7.16 (1.04) | 7.16 (1.00) | 0.98 |
| Sleep duration, seld reported |  |  |  |  |
| <7 hours | 8261 (23 %) | 3920 (24 %) | 4341 (23 %) | 0.0045 |
| 7-9 hours | 24536 (69 %) | 11296 (68 %) | 13240 (70 %) |  |
| >9 hours | 2480 (7 %) | 1237 (7 %) | 1243 (7 %) |  |
| Pain intensity, NRS (0-10) |  |  |  |  |
| Mean (SD) | 3.70 (2.58) | 3.74 (2.59) | 3.66 (2.57) | 0.0797 |
| Depression (PHQ-9, 0-27) |  |  |  |  |
| Mean (SD) | 5.19 (6.98) | 5.41 (7.19) | 5.00 (6.79) | <0.001 |
| Anxiety (GAD-7, 0-21) |  |  |  |  |
| Mean (SD) | 1.80 (3.05) | 1.79 (3.07) | 1.80 (3.02) | 0.989 |
| Neuroticism, 0-12 |  |  |  |  |
| Mean (SD) | 3.23 (2.96) | 3.22 (2.96) | 3.25 (2.97) | 0.57 |
| Brain-fog (SSS) |  |  |  |  |
| Mean (SD) | 1.39 (0.575) | 1.40 (0.581) | 1.39 (0.570) | 0.12 |
| Fatigue Severity Scale (FSS) |  |  |  |  |
| Mean (SD) | 18.6 (13.2) | 18.9 (13.4) | 18.3 (13.0) | <0.001 |
| Doleur Neuropathique 4 (0-7) |  |  |  |  |
| Mean (SD) | 1.16 (1.39) | 1.21 (1.43) | 1.12 (1.36) | <0.001 |
| Opioid use | 1.13% | 1.15% | 1.12% | 0.969 |
| Tricyclic Antidepressant use | 1.53% | 1.70% | 1.39% | 0.0547 |
| Gabapentinoid use | 0.91% | 0.98% | 0.85% | 0.411 |
| Baseline executive function, Z |  |  |  |  |
| Mean (SD) | 0.00249 (0.999) | -0.0715 (1.08) | 0.0672 (0.922) | <0.001 |

At both baseline and follow-up, participants with chronic pain at baseline consistently performed worse across the three cognitive tasks compared to those without chronic pain. On average, individuals with chronic pain scored 1 to 2 percentile ranks lower than those without chronic pain, indicating a modest but statistically significant difference in cognitive performance. There was a notable ceiling effect evident on the SDS.

**Table S5. Cognitive test scores at baseline and follow-up for participants with and without chronic pain. All results adjusted for age at the time of the test**.

Comparisons between groups were conducted using two-sample t-tests. The TMT results indicate the difference in time taken to complete the alphanumeric and numeric courses, with higher values reflecting slower processing speed. SDS measures the proportion of correct responses, and the matrix pattern test assesses abstract reasoning accuracy. Chronic pain at baseline was consistently associated with lower performance, particularly on the TMT and matrix pattern test, highlighting a potential impact of chronic pain on executive function abilities. TMT, trail making test. SDS, digit-symbol substitution test. SD, standard deviation.

| **Baseline** | | | | | **Follow-up** | | | | |
| --- | --- | --- | --- | --- | --- | --- | --- | --- | --- |
|  | **Total** | **No Chronic pain** | **Chronic pain** | **P-value** |  | **Total** | **No Chronic pain** | **Chronic pain** | **P-value** |
|  | (N=35423) | (N=16654) | (N=18769) |  |  | (N=18898) | (N=8876) | (N=10022) |  |
| TMT, seconds |  |  |  |  | TMT, seconds |  |  |  |  |
| Mean (SD) | 34.3 (23.5) | 34.0 (23.4) | 34.6 (23.6) | 0.0308 | Mean (SD) | 30.8 (19.1) | 30.4 (20.4) | 31.2 (18.0) | 0.0186 |
| TMT, centile rank |  |  |  |  | TMT, centile rank |  |  |  |  |
| Mean (SD) | 50.1 (28.8) | 50.6 (28.9) | 49.6 (28.8) | 0.00504 | Mean (SD) | 50.1 (28.9) | 50.9 (28.9) | 49.4 (28.9) | 0.00326 |
| SDS, proportion correct |  |  |  |  | SDS, proportion correct |  |  |  |  |
| Mean (SD) | 0.954 (0.0934) | 0.956 (0.0907) | 0.952 (0.0957) | <0.001 | Mean (SD) | 0.964 (0.0858) | 0.966 (0.0827) | 0.963 (0.0885) | 0.0193 |
| SDS, centile rank |  |  |  |  | SDS, centile rank |  |  |  |  |
| Mean (SD) | 50.0 (28.8) | 50.6 (28.6) | 49.5 (29.1) | 0.00149 | Mean (SD) | 50.0 (28.9) | 50.5 (28.6) | 49.6 (29.1) | 0.0984 |
| Matrix pattern test, proportion correct | |  |  |  | Matrix pattern test, proportion correct | |  |  |  |
| Mean (SD) | 0.597 (0.166) | 0.604 (0.165) | 0.590 (0.167) | <0.001 | Mean (SD) | 0.708 (0.181) | 0.716 (0.180) | 0.701 (0.182) | <0.001 |
| Matrix pattern test, centile rank | |  |  |  | Matrix pattern test, centile rank | |  |  |  |
| Mean (SD) | 50.0 (28.9) | 51.3 (28.8) | 48.9 (28.9) | <0.001 | Mean (SD) | 50.0 (28.8) | 51.3 (28.7) | 48.9 (28.9) | <0.001 |


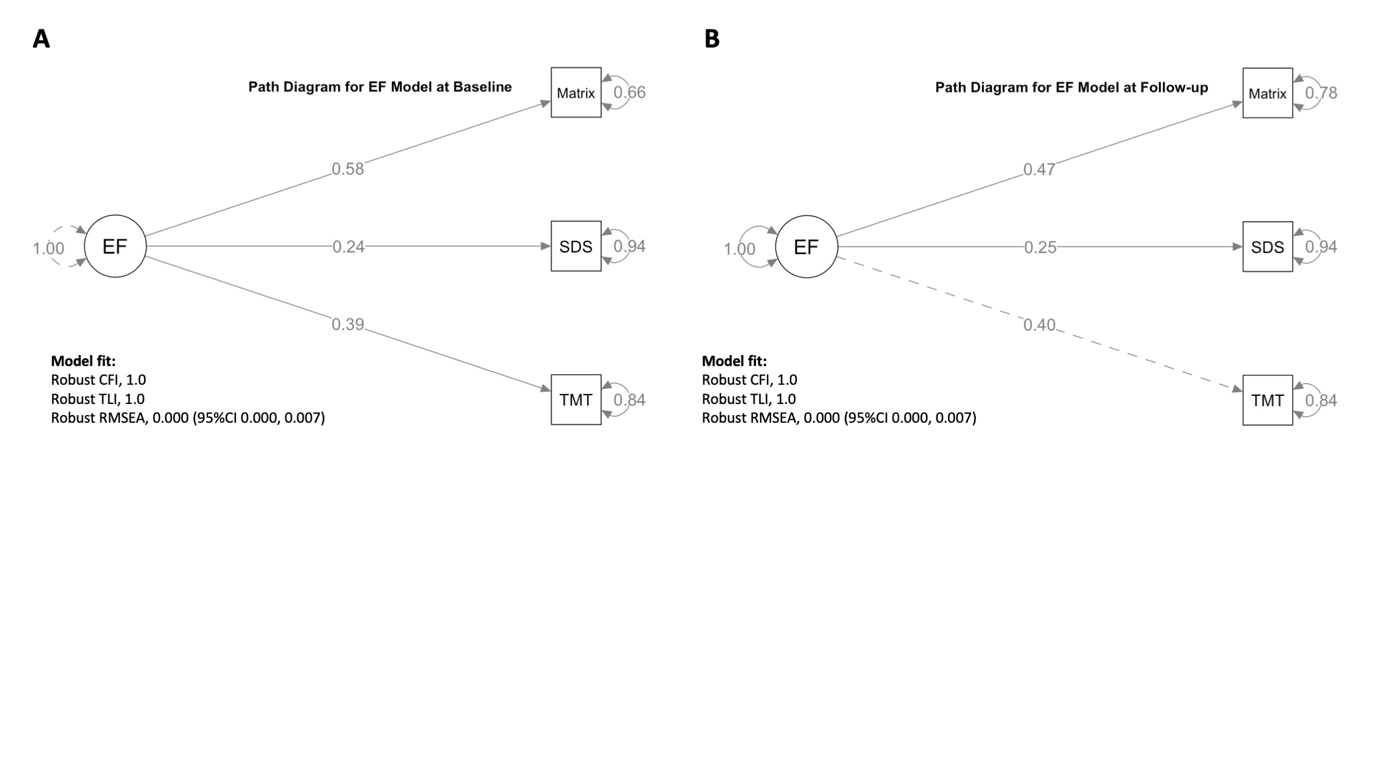


**Figure S2. Path diagrams for latent variables for executive function at baseline**

Standardised loadings and residual variances displayed. Residual variance reflects the proportion of variance in an observed variable unexplained by the latent factor; lower residual variance indicates stronger alignment with the latent construct. EF, executive function. Matrix, matrix pattern recognition test. SDS, digit-symbol substitution test. TMT, trail-making test. CFI, comparative fit index. TLI, Tucker-Lewis index. RMSEA, root mean square error of approximation. 95%CI, 95% confidence interval.

# Appendix C: Cross-sectional analysis: detailed regression results & diagnostics

**Table S6. Cross-sectional relationship between fibromyalgia index (FMI) score and executive function, with chronic pain interaction.**

Linear regression beta coefficients (Estimates), 95% confidence intervals (95% CI), and p-values are shown for two models predicting executive function (age-adjusted Z-score). The exposure of interest if the fibromyalgia index (FMI), which is a on a scale of 0 to 31. Model 1 adjusts for age, sex, and assessment order. Model 2 includes additional adjustments for sociodemographic factors (education level, Townsend deprivation index, ethnicity, body mass index, and smoking status). Interaction terms (e.g., FMI:Chronic pain) assess the moderating effect of chronic pain on the relationship between nociplastic pain severity and executive function. All continuous variables are mean-centred. `fup_cog0_eop` is the time in years between baseline cognitive assessment at the imaging visit, and response to the online pain questionnaire. Due to non-linearity, this was fitted with a polynomial term. The outcome is age-standardised executive function derived from confirmatory factor analysis with the Trail Making Test, Digit Symbol Substitution Test and Matrix Pattern Test (see methods section), and is on a standardised scale with a mean of 0 and standard deviation of 1.

|  | **Model 1** | | | | **Model 2** | | | |
| --- | --- | --- | --- | --- | --- | --- | --- | --- |
| **Predictor** | **Estimate** | **95% CI Lower** | **95% CI Upper** | **P** | **Estimate** | **95% CI Lower** | **95% CI Upper** | **P** |
| (Intercept) | -0.009 | -0.032 | 0.013 | 0.401 | -0.806 | -0.874 | -0.738 | 0.000 |
| FMI:No Chronic pain | 0.005 | -0.003 | 0.013 | 0.213 | 0.007 | 0.000 | 0.015 | 0.054 |
| FMI:Chronic pain | -0.027 | -0.031 | -0.023 | 0.000 | -0.018 | -0.021 | -0.014 | 0.000 |
| Chronic Pain | -0.033 | -0.058 | -0.008 | 0.010 | -0.017 | -0.041 | 0.007 | 0.156 |
| Male sex | 0.113 | 0.092 | 0.134 | 0.000 | 0.122 | 0.101 | 0.142 | 0.000 |
| Age | -0.003 | -0.005 | -0.002 | 0.000 | -0.003 | -0.004 | -0.001 | 0.000 |
| University Degree |  |  |  |  | 0.477 | 0.456 | 0.497 | 0.000 |
| TDI |  |  |  |  | -0.018 | -0.022 | -0.014 | 0.000 |
| White ethnicity |  |  |  |  | 0.543 | 0.478 | 0.607 | 0.000 |
| BMI |  |  |  |  | -0.007 | -0.010 | -0.005 | 0.000 |
| Current Smoker |  |  |  |  | -0.105 | -0.165 | -0.044 | 0.001 |
| poly(fup_cog0_eop, 2)1 | -12.221 | -14.169 | -10.272 | 0.000 | -11.373 | -13.255 | -9.491 | 0.000 |
| poly(fup_cog0_eop, 2)2 | 3.475 | 1.524 | 5.427 | 0.000 | 3.170 | 1.282 | 5.058 | 0.001 |

**Table S7. Cross-sectional relationship between fibromyalgia index (FMI) score and executive function rank, with chronic pain interaction.**

Linear regression beta coefficients (Estimates), 95% confidence intervals (95% CI), and p-values are shown for two models predicting executive function (age-adjusted centile rank). The exposure of interest if the fibromyalgia index (FMI), which is a on a scale of 0 to 31. Model 1 adjusts for age, sex, and assessment order. Model 2 includes additional adjustments for sociodemographic factors (education level, Townsend deprivation index, ethnicity, body mass index, and smoking status). Interaction terms (e.g., FMI:Chronic pain) assess the moderating effect of chronic pain on the relationship between nociplastic pain severity and executive function. All continuous variables are mean-centred. `fup_cog0_eop` is the time in years between baseline cognitive assessment at the imaging visit, and response to the online pain questionnaire. Due to non-linearity, this was fitted with a polynomial term. The outcome is age-standardised executive function centile rank derived from confirmatory factor analysis with the Trail Making Test, Digit Symbol Substitution Test and Matrix Pattern Test (see methods section).

|  | **Model 1** | | | | **Model 2** | | | |
| --- | --- | --- | --- | --- | --- | --- | --- | --- |
| **Predictor** | **Estimate** | **95% CI Lower** | **95% CI Upper** | **P** | **Estimate** | **95% CI Lower** | **95% CI Upper** | **P** |
| (Intercept) | 49.61 | 48.97 | 50.25 | 0.000 | 27.98 | 26.02 | 29.94 | 0.000 |
| FMI:No Chronic pain | 0.17 | -0.06 | 0.39 | 0.152 | 0.24 | 0.02 | 0.46 | 0.031 |
| FMI:Chronic pain | -0.77 | -0.87 | -0.66 | 0.000 | -0.49 | -0.59 | -0.39 | 0.000 |
| Chronic Pain | -1.18 | -1.90 | -0.46 | 0.001 | -0.70 | -1.39 | -0.01 | 0.048 |
| Male sex | 3.80 | 3.20 | 4.41 | 0.000 | 4.10 | 3.52 | 4.69 | 0.000 |
| Age | 0.03 | -0.01 | 0.08 | 0.104 | 0.06 | 0.02 | 0.10 | 0.006 |
| University Degree |  |  |  |  | 14.31 | 13.72 | 14.89 | 0.000 |
| TDI |  |  |  |  | -0.42 | -0.53 | -0.31 | 0.000 |
| White ethnicity |  |  |  |  | 13.96 | 12.10 | 15.82 | 0.000 |
| BMI |  |  |  |  | -0.25 | -0.31 | -0.18 | 0.000 |
| Current Smoker |  |  |  |  | -3.84 | -5.59 | -2.09 | 0.000 |
| poly(fup_cog0_eop, 2)1 | -415.34 | -471.52 | -359.16 | 0.000 | -387.46 | -441.61 | -333.31 | 0.000 |
| poly(fup_cog0_eop, 2)2 | 86.00 | 29.74 | 142.25 | 0.003 | 83.00 | 28.67 | 137.34 | 0.003 |

**
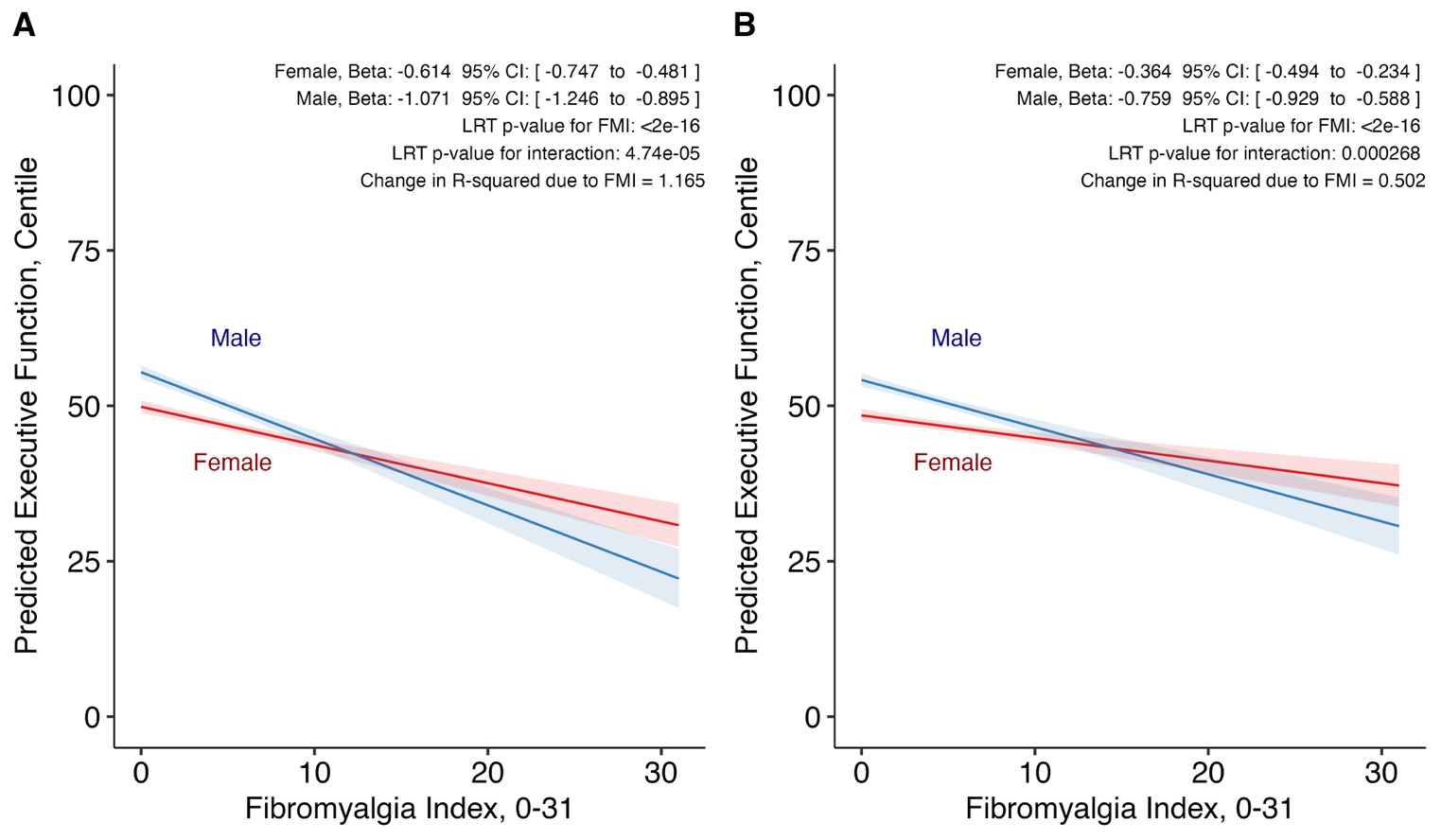
**

**Figure S3. Sex Differences in the Association Between Fibromyalgia Index (FMI) and Executive Function (EF)**

(A and B) Stronger Negative Association Between FMI and EF in Males Compared to Females.
Panels depict predicted EF centile scores across FMI levels, stratified by sex. Panel A adjusts for age, sex, and assessment order, while Panel B includes additional adjustments for ethnicity, socioeconomic status, education, body mass index (BMI), and tobacco use.

Lines represent estimated associations, with shaded regions indicating 95% confidence intervals. A significant interaction between FMI and sex is observed, with males showing a stronger negative association between FMI and EF than females, as indicated by the interaction P-values from likelihood ratio tests (LRT). These findings suggest that increasing FMI scores are associated with greater EF reductions in males.

Note: Centile scores represent an individual’s performance relative to the general population, with a score of 50 corresponding to the median, higher scores indicating better EF, and lower scores indicating poorer EF.

**Table S8. Cross-sectional relationship between fibromyalgia index (FMI) score and executive function, with sex interaction.**

Linear regression beta coefficients (Estimates), 95% confidence intervals (95% CI), and p-values are shown for two models predicting executive function (age-adjusted Z-score) among participants with chronic pain. The exposure of interest if the fibromyalgia index (FMI), which is a on a scale of 0 to 31. Model 1 adjusts for age, sex, and assessment order. Model 2 includes additional adjustments for sociodemographic factors (education level, Townsend deprivation index, ethnicity, body mass index, and smoking status). Interaction terms (e.g., FMI:Female) assess the moderating effect of chronic pain on the relationship between nociplastic pain severity and executive function. All continuous variables are mean-centred. `fup_cog0_eop` is the time in years between baseline cognitive assessment at the imaging visit, and response to the online pain questionnaire. Due to non-linearity, this was fitted with a polynomial term. The outcome is age-standardised executive function derived from confirmatory factor analysis with the Trail Making Test, Digit Symbol Substitution Test and Matrix Pattern Test (see methods section), and is on a standardised scale with a mean of 0 and standard deviation of 1.

|  | **Model 1** | | | | **Model 2** | | | |
| --- | --- | --- | --- | --- | --- | --- | --- | --- |
| **Predictor** | **Estimate** | **95% CI Lower** | **95% CI Upper** | **P** | **Estimate** | **95% CI Lower** | **95% CI Upper** | **P** |
| (Intercept) | 0.057 | 0.037 | 0.076 | 0.000 | -0.725 | -0.792 | -0.658 | 0.000 |
| FMI:Female | -0.019 | -0.023 | -0.015 | 0.000 | -0.011 | -0.015 | -0.007 | 0.000 |
| FMI:Male | -0.026 | -0.031 | -0.021 | 0.000 | -0.017 | -0.022 | -0.012 | 0.000 |
| Chronic Pain | 0.003 | -0.020 | 0.026 | 0.786 | 0.011 | -0.011 | 0.033 | 0.340 |
| Male sex | -0.111 | -0.132 | -0.090 | 0.000 | -0.120 | -0.141 | -0.100 | 0.000 |
| Age | -0.003 | -0.005 | -0.002 | 0.000 | -0.003 | -0.004 | -0.001 | 0.000 |
| University Degree |  |  |  |  | 0.478 | 0.458 | 0.498 | 0.000 |
| TDI |  |  |  |  | -0.018 | -0.022 | -0.014 | 0.000 |
| White ethnicity |  |  |  |  | 0.546 | 0.482 | 0.611 | 0.000 |
| BMI |  |  |  |  | -0.007 | -0.010 | -0.005 | 0.000 |
| Current Smoker |  |  |  |  | -0.106 | -0.167 | -0.045 | 0.001 |
| poly(fup_cog0_eop, 2)1 | -12.265 | -14.215 | -10.316 | 0.000 | -11.404 | -13.287 | -9.522 | 0.000 |
| poly(fup_cog0_eop, 2)2 | 3.489 | 1.537 | 5.442 | 0.000 | 3.181 | 1.292 | 5.070 | 0.001 |

**Table S9. Cross-sectional relationship between fibromyalgia index (FMI) score and executive function, with sex interaction.**

Linear regression beta coefficients (Estimates), 95% confidence intervals (95% CI), and p-values are shown for two models predicting executive function (age-adjusted centile rank) among participants with chronic pain. The exposure of interest if the fibromyalgia index (FMI), which is a on a scale of 0 to 31. Model 1 adjusts for age, sex, and assessment order. Model 2 includes additional adjustments for sociodemographic factors (education level, Townsend deprivation index, ethnicity, body mass index, and smoking status). Interaction terms (e.g., FMI:Female) assess the moderating effect of chronic pain on the relationship between nociplastic pain severity and executive function. All continuous variables are mean-centred. `fup_cog0_eop` is the time in years between baseline cognitive assessment at the imaging visit, and response to the online pain questionnaire. Due to non-linearity, this was fitted with a polynomial term. The outcome is age-standardised executive function derived from confirmatory factor analysis with the Trail Making Test, Digit Symbol Substitution Test and Matrix Pattern Test (see methods section).

|  | **Model 1** | | | | **Model 2** | | | |
| --- | --- | --- | --- | --- | --- | --- | --- | --- |
| **Predictor** | **Estimate** | **95% CI Lower** | **95% CI Upper** | **P** | **Estimate** | **95% CI Lower** | **95% CI Upper** | **P** |
| (Intercept) | 52.032 | 51.469 | 52.596 | 0.000 | 30.882 | 28.950 | 32.815 | 0.000 |
| FMI:Female | -0.506 | -0.622 | -0.390 | 0.000 | -0.278 | -0.392 | -0.165 | 0.000 |
| FMI:Male | -0.772 | -0.921 | -0.623 | 0.000 | -0.500 | -0.644 | -0.355 | 0.000 |
| Chronic Pain | -0.129 | -0.795 | 0.537 | 0.705 | 0.127 | -0.515 | 0.769 | 0.699 |
| Male sex | -3.725 | -4.332 | -3.118 | 0.000 | -4.047 | -4.638 | -3.457 | 0.000 |
| Age | 0.028 | -0.013 | 0.069 | 0.179 | 0.052 | 0.012 | 0.092 | 0.011 |
| University Degree |  |  |  |  | 14.336 | 13.753 | 14.918 | 0.000 |
| TDI |  |  |  |  | -0.419 | -0.526 | -0.312 | 0.000 |
| White ethnicity |  |  |  |  | 14.060 | 12.199 | 15.920 | 0.000 |
| BMI |  |  |  |  | -0.253 | -0.320 | -0.187 | 0.000 |
| Current Smoker |  |  |  |  | -3.870 | -5.625 | -2.115 | 0.000 |
| poly(fup_cog0_eop, 2)1 | -416.527 | -472.741 | -360.312 | 0.000 | -388.247 | -442.422 | -334.073 | 0.000 |
| poly(fup_cog0_eop, 2)2 | 86.321 | 30.028 | 142.613 | 0.003 | 83.241 | 28.882 | 137.600 | 0.003 |

**Table S10. No evidence of collinearity present in model.**

GVIF used to account for variables with multiple degrees of freedom. GVIF, generalised variance inflation factor. Df, degrees of freedom.

|  | **GVIF** | **Df** | **GVIF^(1/(2*Df))** |
| --- | --- | --- | --- |
| Chronic pain | 1.45336921 | 1 | 1.20555763 |
| Male sex | 1.05426982 | 1 | 1.02677642 |
| Baseline age | 1.05984808 | 1 | 1.02948923 |
| University degree | 1.02786145 | 1 | 1.01383502 |
| Townsend Deprivation Index (centred) | 1.03277905 | 1 | 1.01625738 |
| White ethnicity | 1.01687251 | 1 | 1.00840097 |
| Body Mass Index (centred) | 1.06124767 | 1 | 1.03016876 |
| Current smoker | 1.0112869 | 1 | 1.00562762 |
| poly(fup_cog0_eop, 2) | 1.02213037 | 2 | 1.00548726 |
| fmi_centered:cp_eop_bin | 1.52822514 | 2 | 1.11185163 |

**Table S11. Model Fit Indices for Factorial Invariance Testing of Executive Function Across Time Points.**

Fit indices for the configural, metric, partial scalar, and scalar models assessing factorial invariance of executive function across time points. The configural, metric, and partial scalar models demonstrated acceptable fit, supporting partial invariance (intercept of TMT allowed to vary) of executive function measures over time. However, the scalar model showed poor fit, indicating that full scalar invariance does not hold. Change in CFI or TLI 0.01, RMSEA ≥0.015, and SRMR ≤0.03 between models were considered indicators of factorial invariance. Df, degrees of freedom. CFI, confirmatory factor index. TLI, Tucker Lewis Index. RMSEA, root mean square error of approximation. SRMR, standardised root mean square error residual.

|  | **Parameters (N)** | **Chi-Square** | **Df** | **CFI (robust)** | **TLI (robust)** | **RMSEA** | **SRMR** |
| --- | --- | --- | --- | --- | --- | --- | --- |
| Configural | 20 | 119.108 | 7 | 0.982 | 0.961 | 0.040 | 0.021 |
| Metric | 19 | 127.996 | 8 | 0.980 | 0.963 | 0.039 | 0.021 |
| Partial Scalar | 18 | 133.011 | 9 | 0.980 | 0.966 | 0.037 | 0.022 |
| Scalar | 17 | 6233.461 | 10 | 0.000 | -0.565 | 0.249 | 0.149 |


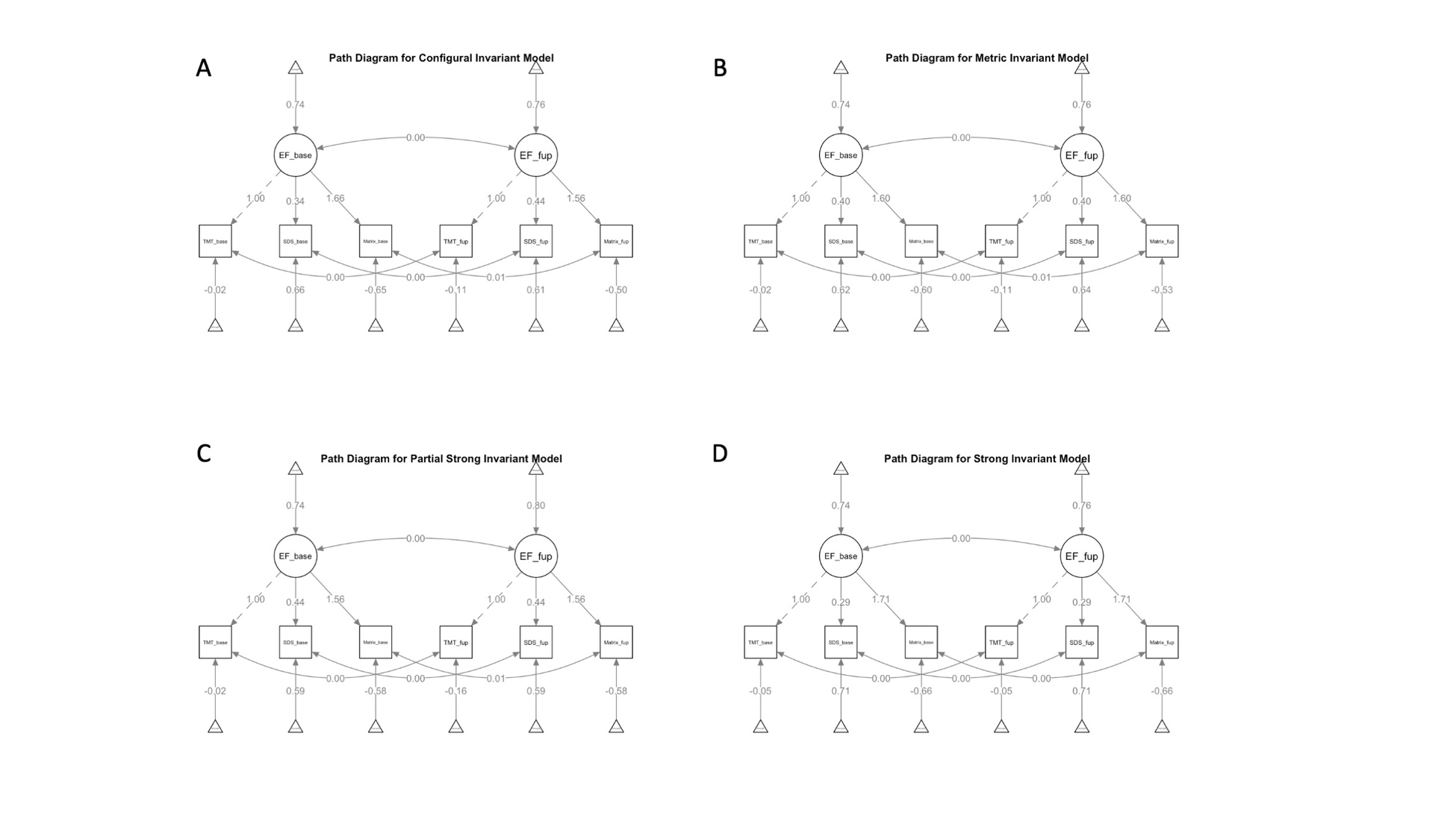


**Figure S4. Partial scalar invariance exists over time for executive function for UK Biobank participants with chronic time.**

Path diagrams illustrate the factorial invariance testing for executive function (EF) across time points (baseline, EF_base; follow-up, EF_fup). Panel A shows the configural invariance model, which establishes the baseline structure. Panel B depicts the metric invariance model, where factor loadings are constrained to be equal across time points. Panel C represents the partial scalar invariance model, with both loadings and intercepts partially constrained (intercept for TMT permitted to vary), indicating partial invariance. Panel D illustrates the strong invariance model, where full scalar invariance is tested. The effects coding method of scaling is used, where the loadings are constrained to equal three, and the intercepts to equal zero. Only partial scalar invariance was achieved, as seen in the fit indices. These findings suggest that while some aspects of the EF construct are stable over time, full scalar invariance does not hold.

# Appendix D: Longitudinal CFA

**Table S12. Longitudinal SEM analysis of the indirect and direct effects of nociplastic pain severity (FMI) on executive function.**

This table presents standardised parameter estimates, 95% confidence intervals (CI), standard errors (SE), z-values, and p-values for indirect pathways (via baseline EF), the total effect, and the direct effect of FMI on follow-up EF. Negative estimates indicate associations with lower executive function scores. EF_base, baseline executive function. EF_fup, follow-up executive function. FMI, fibromyalgia index.

| **Path** | **Predictor** | **Estimate** | **SE** | **z-value** | **p-value** | **CI Lower** | **CI Upper** |
| --- | --- | --- | --- | --- | --- | --- | --- |
| **Unadjusted** |  |  |  |  |  |  |  |
| EF_fup | FMI | -0.043 | 0.017 | -2.578 | 0.0099 | -0.075 | -0.010 |
| EF_base | FMI | -0.208 | 0.019 | -11.134 | 0.0000 | -0.245 | -0.172 |
| indirect (via EF_base) | a1*b1 | -0.153 | 0.015 | -10.455 | 0.0000 | -0.182 | -0.125 |
| direct | c0 | -0.043 | 0.017 | -2.578 | 0.0099 | -0.075 | -0.010 |
| total | a1*b1+c0 | -0.196 | 0.017 | -11.599 | 0.0000 | -0.229 | -0.163 |
| **Minimally adjusted** |  |  |  |  |  |  |  |
| EF_fup | FMI | -0.042 | 0.017 | -2.536 | 0.0112 | -0.074 | -0.010 |
| EF_base | FMI | -0.191 | 0.019 | -10.086 | 0.0000 | -0.228 | -0.154 |
| indirect (via EF_base) | a1*b1 | -0.141 | 0.015 | -9.556 | 0.0000 | -0.170 | -0.112 |
| direct | c0 | -0.042 | 0.017 | -2.536 | 0.0112 | -0.074 | -0.010 |
| total | direct+indirect | -0.183 | 0.017 | -10.549 | 0.0000 | -0.217 | -0.149 |
| **Fully adjusted** |  |  |  |  |  |  |  |
| EF_fup | FMI | -0.021 | 0.017 | -1.212 | 0.2255 | -0.055 | 0.013 |
| EF_base | FMI | -0.110 | 0.016 | -6.734 | 0.0000 | -0.142 | -0.078 |
| indirect (via EF_base) | a1*b1 | -0.107 | 0.017 | -6.417 | 0.0000 | -0.140 | -0.074 |
| direct | c0 | -0.021 | 0.017 | -1.212 | 0.2255 | -0.055 | 0.013 |
| total | indirect+direct | -0.128 | 0.018 | -7.165 | 0.0000 | -0.163 | -0.093 |

# Appendix E. Mediation analyses

**Table S13. Mediation analysis of the indirect and direct effects of SPACE symptom domains on the association between Fibromyalgia Index (FMI) score and executive function.**

This table presents standardised parameter estimates, 95% confidence intervals (CI), standard errors (SE), z-values, and p-values for indirect pathways (baseline EF [cog], sleep, pain, depression, anxiety, brain-fog, and fatigue), the total effect, the combined indirect effect of all SPACE symptoms, and the direct effect of FMI on EF. Negative estimates indicate associations with lower executive function scores. Indirect effects on follow-up EF are indicated by paths annotated by “1”, while those on baseline EF are indicated by “0”. EF_base, baseline executive function. EF_fup, follow-up executive function. FMI, fibromyalgia index.

| **Path** | **Predictor** | **Estimate** | **SE** | **z-value** | **p-value** | **CI Lower** | **CI Upper** |
| --- | --- | --- | --- | --- | --- | --- | --- |
| EF_fup | FMI | 0.009 | 0.021 | 0.446 | 0.65545 | -0.032 | 0.051 |
| EF_base | FMI | -0.082 | 0.027 | -3.017 | 0.00255 | -0.136 | -0.029 |
| cog | a0*b0 | -0.064 | 0.021 | -2.977 | 0.00291 | -0.106 | -0.022 |
| sleep1 | a1*b1 | 0.001 | 0.002 | 0.526 | 0.59865 | -0.003 | 0.006 |
| pain1 | a2*b2 | 0.000 | 0.005 | 0.078 | 0.93803 | -0.010 | 0.011 |
| depression1 | a3*b3 | -0.005 | 0.012 | -0.371 | 0.71086 | -0.029 | 0.020 |
| anxiety1 | a4*b4 | -0.010 | 0.005 | -1.824 | 0.06817 | -0.020 | 0.001 |
| brainfog1 | a5*b5 | -0.006 | 0.011 | -0.527 | 0.59799 | -0.027 | 0.016 |
| fatigue1 | a6*b6 | -0.018 | 0.013 | -1.401 | 0.16126 | -0.043 | 0.007 |
| total1 | c0+cog+sleep1+pain1+depression1+anxiety1+brainfog1+fatigue1 | -0.091 | 0.023 | -3.948 | 0.00008 | -0.136 | -0.046 |
| space1 | sleep1+pain1+depression1+anxiety1+brainfog1+fatigue1 | -0.036 | 0.015 | -2.350 | 0.01878 | -0.067 | -0.006 |
| direct1 | c0 | 0.009 | 0.021 | 0.446 | 0.65545 | -0.032 | 0.051 |
| sleep0 | a1*e1 | -0.013 | 0.003 | -4.580 | 0.00000 | -0.019 | -0.008 |
| pain0 | a2*e2 | -0.030 | 0.006 | -5.112 | 0.00000 | -0.042 | -0.019 |
| depression0 | a3*e3 | 0.045 | 0.015 | 2.952 | 0.00316 | 0.015 | 0.074 |
| anxiety0 | a4*e4 | -0.030 | 0.007 | -4.253 | 0.00002 | -0.043 | -0.016 |
| brainfog0 | a5*e5 | -0.004 | 0.014 | -0.321 | 0.74830 | -0.031 | 0.022 |
| fatigue0 | a6*e6 | -0.004 | 0.016 | -0.238 | 0.81200 | -0.036 | 0.028 |
| total0 | a0+sleep0+pain0+depression0+anxiety0+brainfog0+fatigue0 | -0.119 | 0.017 | -6.836 | 0.00000 | -0.154 | -0.085 |
| space0 | sleep0+pain0+depression0+anxiety0+brainfog0+fatigue0 | -0.037 | 0.020 | -1.811 | 0.07015 | -0.077 | 0.003 |
| direct0 | a0 | -0.082 | 0.027 | -3.017 | 0.00255 | -0.136 | -0.029 |

**Table S14. Mediation analysis of the indirect and direct effects of pain characteristics on the association between Fibromyalgia Index (FMI) score and executive function.**

This table presents standardised parameter estimates, 95% confidence intervals (CI), standard errors (SE), z-values, and p-values for indirect pathways (baseline EF [cog], pain severity, widespread pain [wpi], and neuropathic pain), the total effect, the combined indirect effect of all pain characteristics, and the direct effect of FMI on EF. Negative estimates indicate associations with lower executive function scores. Indirect effects on follow-up EF are indicated by paths annotated by “1”, while those on baseline EF are indicated by “0”. EF_base, baseline executive function. EF_fup, follow-up executive function. FMI, fibromyalgia index.

| **Path** | **Predictor** | **Estimate** | **SE** | **z-value** | **p-value** | **CI Lower** | **CI Upper** |
| --- | --- | --- | --- | --- | --- | --- | --- |
| EF_fup | fmi.z | -0.038 | 0.029 | -1.305 | 0.19198 | -0.096 | 0.019 |
| EF_base | fmi.z | -0.128 | 0.034 | -3.711 | 0.00021 | -0.195 | -0.060 |
| cog | a0*b0 | -0.099 | 0.027 | -3.683 | 0.00023 | -0.152 | -0.046 |
| pain1 | a1*b1 | 0.001 | 0.005 | 0.170 | 0.86476 | -0.010 | 0.011 |
| wpi1 | a2*b2 | 0.018 | 0.024 | 0.742 | 0.45811 | -0.029 | 0.065 |
| neuropathic1 | a3*b3 | -0.008 | 0.005 | -1.433 | 0.15182 | -0.018 | 0.003 |
| total1 | c0+cog+pain1+wpi1+neuropathic1 | -0.126 | 0.028 | -4.471 | 0.00001 | -0.181 | -0.071 |
| indirect1 | pain1+wpi1+neuropathic1 | 0.011 | 0.024 | 0.451 | 0.65201 | -0.037 | 0.059 |
| direct1 | c0 | -0.038 | 0.029 | -1.305 | 0.19198 | -0.096 | 0.019 |
| pain0 | a1*e1 | -0.027 | 0.006 | -4.192 | 0.00003 | -0.039 | -0.014 |
| wpi0 | a2*e2 | 0.069 | 0.029 | 2.381 | 0.01728 | 0.012 | 0.126 |
| neuropathic0 | a3*e3 | -0.034 | 0.007 | -5.109 | 0.00000 | -0.047 | -0.021 |
| total0 | a0+pain0+wpi0+neuropathic0 | -0.120 | 0.018 | -6.776 | 0.00000 | -0.154 | -0.085 |
| indirect0 | pain0+wpi0+neuropathic0 | 0.008 | 0.030 | 0.273 | 0.78507 | -0.050 | 0.066 |
| direct0 | a0 | -0.128 | 0.034 | -3.711 | 0.00021 | -0.195 | -0.060 |

**Table S15. Mediation analysis of the indirect and direct effects of self-reported analgesia use on the association between Fibromyalgia Index (FMI) score and executive function.**

This table presents standardised parameter estimates, 95% confidence intervals (CI), standard errors (SE), z-values, and p-values for indirect pathways (baseline EF [cog], self-reported opioid, tricyclic antidepressant [tca], and gabapentinoid [gaba] use), the total effect, the combined indirect effect of all analgesia use, and the direct effect of FMI on EF. Negative estimates indicate associations with lower executive function scores. Indirect effects on follow-up EF are indicated by paths annotated by “1”, while those on baseline EF are indicated by “0”. EF_base, baseline executive function. EF_fup, follow-up executive function. FMI, fibromyalgia index.

| **Path** | **Predictor** | **Estimate** | **SE** | **z-value** | **p-value** | **CI Lower** | **CI Upper** |
| --- | --- | --- | --- | --- | --- | --- | --- |
| EF_fup | FMI | -0.032 | 0.016 | -2.026 | 0.04276 | -0.064 | -0.001 |
| EF_base | FMI | -0.112 | 0.018 | -6.178 | 0.00000 | -0.147 | -0.076 |
| cog | a0*b0 | -0.087 | 0.014 | -6.041 | 0.00000 | -0.115 | -0.059 |
| opioid1 | a1*b1 | 0.003 | 0.002 | 1.481 | 0.13853 | -0.001 | 0.007 |
| tca1 | a2*b2 | 0.002 | 0.002 | 1.335 | 0.18172 | -0.001 | 0.005 |
| gaba1 | a3*b3 | 0.000 | 0.002 | -0.002 | 0.99878 | -0.004 | 0.004 |
| total1 | c0+cog+opioid1+tca1+gaba1 | -0.114 | 0.017 | -6.618 | 0.00000 | -0.148 | -0.080 |
| indirect1 | opioid1+tca1+gaba1 | 0.005 | 0.003 | 1.758 | 0.07869 | -0.001 | 0.011 |
| direct1 | c0 | -0.032 | 0.016 | -2.026 | 0.04276 | -0.064 | -0.001 |
| opioid0 | a1*e1 | -0.002 | 0.003 | -0.707 | 0.47953 | -0.007 | 0.003 |
| tca0 | a2*e2 | -0.004 | 0.002 | -2.011 | 0.04437 | -0.008 | 0.000 |
| gaba0 | a3*e3 | -0.003 | 0.002 | -1.008 | 0.31341 | -0.007 | 0.002 |
| total0 | a0+opioid0+tca0+gaba0 | -0.120 | 0.018 | -6.779 | 0.00000 | -0.155 | -0.085 |
| indirect0 | opioid0+tca0+gaba0 | -0.008 | 0.004 | -2.187 | 0.02874 | -0.016 | -0.001 |
| direct0 | a0 | -0.112 | 0.018 | -6.178 | 0.00000 | -0.147 | -0.076 |

# Appendix F. Sensitivity analyses

## Individual cognitive tests


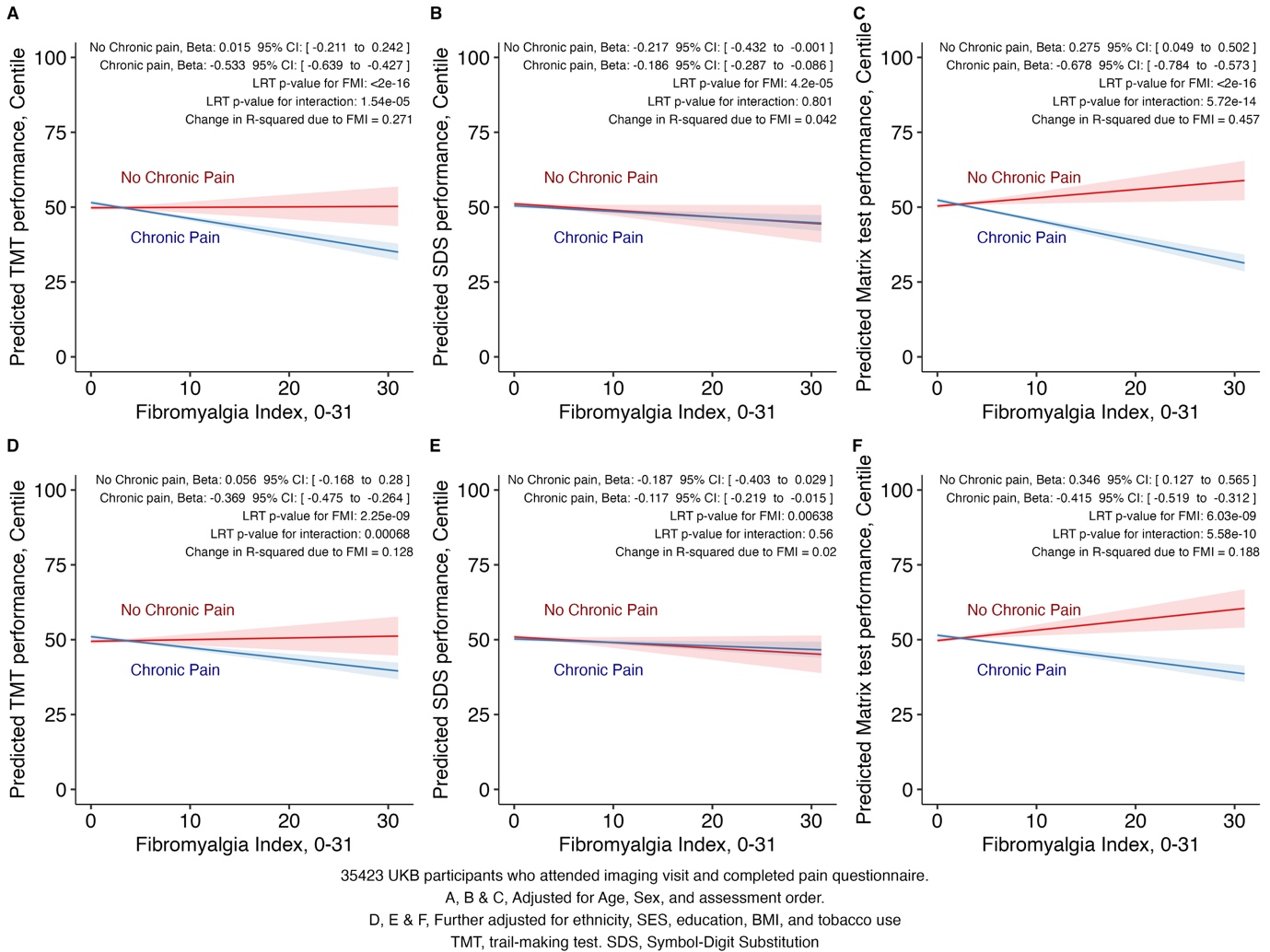
Higher FMI scores were associated with poorer performance on each of the three cognitive tasks among participants with chronic pain, with a significant interaction with chronic pain for the TMT and Matrix pattern test (Figure S5). The strongest effect was seen with the Matrix pattern test (Figure S5 **C&F**), and weakest with the SDS (Figure S5 **B&E**), perhaps reflecting the ceiling effect observed in this latter task.

**Figure S5. Higher fibromyalgia index (FMI) associated with worse performance on all three tests of executive function in adults with chronic pain.**

Panels display predicted cognitive performance scores in centiles for trail-making test (TMT, panels A and D), digit-symbol substitution (SDS, panels B and E), and matrix pattern recognition (Matrix, panels C and F) across FMI scores for participants with and without chronic pain. Panels A, B, and C are adjusted for age, sex, and assessment order, while panels D, E, and F include further adjustments for ethnicity, socioeconomic status, education, body mass index (BMI), and tobacco use. Shaded regions represent 95% confidence intervals. A stronger negative association between FMI and cognitive performance is evident in participants with chronic pain across the TMT and Matrix tasks, as indicated by significant interaction P-values. This suggests that increasing FMI scores are associated with reduced cognitive function specifically among individuals with chronic pain.

**Table S16. Cross-sectional relationship between fibromyalgia index (FMI) score and Matrix Pattern test score, with interaction with chronic pain.**

Linear regression beta coefficients (Estimates), 95% confidence intervals (95% CI), and p-values are shown for two models predicting matrix pattern test (centile rank, where higher values indicate better performance) with an interaction with chronic pain. The exposure of interest is the fibromyalgia index (FMI), which is mean centred and a on a scale of 0 to 31. Model 1 adjusts for age, sex, and assessment order. Model 2 includes additional adjustments for sociodemographic factors (education level, Townsend deprivation index, ethnicity, body mass index, and smoking status). Interaction terms (e.g., FMI:Chronic Pain) assess the moderating effect of chronic pain on the relationship between nociplastic pain severity and matrix pattern test. All continuous variables are mean-centred. `fup_cog0_eop` is the time in years between baseline cognitive assessment at the imaging visit, and response to the online pain questionnaire. Due to non-linearity, this was fitted with a polynomial term.

|  | **Estimate** | **Std. Error** | **t value** | **Pr(>\|t\|)** |
| --- | --- | --- | --- | --- |
| **Minimally-adjusted model** | | |  |  |
| (Intercept) | 49.537 | 0.326 | 152.036 | 0 |
| Chronic Pain | -1.429 | 0.367 | -3.896 | 0.0001 |
| Male Sex | 4.171 | 0.31 | 13.465 | 0 |
| Baseline Age | -0.101 | 0.021 | -4.796 | 0 |
| poly(fup_cog0_eop, 2)1 | -295.429 | 28.715 | -10.288 | 0 |
| poly(fup_cog0_eop, 2)2 | 25.926 | 28.755 | 0.902 | 0.36727 |
| FMI:No Chronic pain | 0.275 | 0.116 | 2.383 | 0.01719 |
| FMI:Chronic pain | -0.678 | 0.054 | -12.561 | 0 |
|  |  |  |  |  |
| **Fully-adjusted model** | |  |  |  |
|  | **Estimate** | **Std. Error** | **t value** | **Pr(>\|t\|)** |
| **(Intercept)** | 29.547 | 1.005 | 29.4 | 0 |
| Chronic Pain | -0.967 | 0.355 | -2.724 | 0.00645 |
| Male Sex | 4.437 | 0.302 | 14.682 | 0 |
| Baseline Age | -0.074 | 0.021 | -3.613 | 0.0003 |
| University Degree | 13.891 | 0.299 | 46.53 | 0 |
| TDI | -0.365 | 0.055 | -6.679 | 0 |
| White ethnicity | 12.493 | 0.954 | 13.097 | 0 |
| BMI | -0.234 | 0.034 | -6.892 | 0 |
| Current smoker | -2.488 | 0.899 | -2.766 | 0.00567 |
| poly(fup_cog0_eop, 2)1 | -268.606 | 27.769 | -9.673 | 0 |
| poly(fup_cog0_eop, 2)2 | 25.297 | 27.863 | 0.908 | 0.36394 |
| FMI:No Chronic pain | 0.346 | 0.112 | 3.092 | 0.00199 |
| FMI:Chronic pain | -0.415 | 0.053 | -7.856 | 0 |

**Table S17. Cross-sectional relationship between fibromyalgia index (FMI) score and Trail Making Test score, with interaction with chronic pain.**

Linear regression beta coefficients (Estimates), 95% confidence intervals (95% CI), and p-values are shown for two models predicting Trail Making Test performance (centile rank, where higher values indicate better performance) with an interaction with chronic pain. The exposure of interest is the fibromyalgia index (FMI), which is mean centred and a on a scale of 0 to 31. Model 1 adjusts for age, sex, and assessment order. Model 2 includes additional adjustments for sociodemographic factors (education level, Townsend deprivation index, ethnicity, body mass index, and smoking status). Interaction terms (e.g., FMI:Chronic Pain) assess the moderating effect of chronic pain on the relationship between nociplastic pain severity and Trail Making test. All continuous variables are mean-centred. `fup_cog0_eop` is the time in years between baseline cognitive assessment at the imaging visit, and response to the online pain questionnaire. Due to non-linearity, this was fitted with a polynomial term.

|  | **Estimate** | **Std. Error** | **t value** | **Pr(>\|t\|)** |
| --- | --- | --- | --- | --- |
| **Minimally-adjusted model** |  |  |  |  |
| (Intercept) | 50.247 | 0.326 | 154.239 | 0.00000 |
| Chronic Pain | -0.235 | 0.367 | -0.641 | 0.52183 |
| Male Sex | 0.823 | 0.310 | 2.658 | 0.00787 |
| Baseline Age | 0.324 | 0.021 | 15.461 | 0.00000 |
| poly(fup_cog0_eop, 2)1 | -377.245 | 28.711 | -13.139 | 0.00000 |
| poly(fup_cog0_eop, 2)2 | 119.387 | 28.750 | 4.153 | 0.00003 |
| fmi_centered:cp_eop_binNo Chronic pain | 0.015 | 0.116 | 0.132 | 0.89502 |
| fmi_centered:cp_eop_binChronic pain | -0.533 | 0.054 | -9.875 | 0.00000 |
|  |  |  |  |  |
| **Fully-adjusted model** |  |  |  |  |
| (Intercept) | 35.852 | 1.026 | 34.957 | 0.00000 |
| Chronic Pain | 0.015 | 0.362 | 0.042 | 0.96620 |
| Male Sex | 0.948 | 0.308 | 3.075 | 0.00211 |
| Baseline Age | 0.331 | 0.021 | 15.821 | 0.00000 |
| University Degree | 8.613 | 0.305 | 28.270 | 0.00000 |
| TDI | -0.363 | 0.056 | -6.507 | 0.00000 |
| White ethnicity | 9.901 | 0.973 | 10.171 | 0.00000 |
| BMI | -0.084 | 0.035 | -2.413 | 0.01583 |
| Current smoker | -3.904 | 0.918 | -4.254 | 0.00002 |
| poly(fup_cog0_eop, 2)1 | -361.493 | 28.338 | -12.756 | 0.00000 |
| poly(fup_cog0_eop, 2)2 | 112.066 | 28.434 | 3.941 | 0.00008 |
| FMI:No Chronic pain | 0.056 | 0.114 | 0.491 | 0.62328 |
| FMI:Chronic pain | -0.369 | 0.054 | -6.848 | 0.00000 |

**Table S18. Cross-sectional relationship between fibromyalgia index (FMI) score and Digit-Symbol Substitution Test score, with interaction with chronic pain.**

Linear regression beta coefficients (Estimates), 95% confidence intervals (95% CI), and p-values are shown for two models predicting Digit-Symbol Substitution Test performance (centile rank, where higher values indicate better performance) with an interaction with chronic pain. The exposure of interest is the fibromyalgia index (FMI), which is mean centred and a on a scale of 0 to 31. Model 1 adjusts for age, sex, and assessment order. Model 2 includes additional adjustments for sociodemographic factors (education level, Townsend deprivation index, ethnicity, body mass index, and smoking status). Interaction terms (e.g., FMI:Chronic Pain) assess the moderating effect of chronic pain on the relationship between nociplastic pain severity and Digit-Symbol Substitution Test. All continuous variables are mean-centred. `fup_cog0_eop` is the time in years between baseline cognitive assessment at the imaging visit, and response to the online pain questionnaire. Due to non-linearity, this was fitted with a polynomial term.

|  | **Estimate** | **Std. Error** | **t value** | **Pr(>\|t\|)** |
| --- | --- | --- | --- | --- |
| **Minimally-adjusted model** | | |  |  |
| (Intercept) | 49.324 | 0.31 | 159.104 | 0 |
| Chronic Pain | -0.559 | 0.349 | -1.601 | 0.10934 |
| Male Sex | 2.025 | 0.295 | 6.871 | 0 |
| Baseline Age | 1.221 | 0.02 | 61.149 | 0 |
| poly(fup_cog0_eop, 2)1 | -528.573 | 27.322 | -19.346 | 0 |
| poly(fup_cog0_eop, 2)2 | -8.021 | 27.359 | -0.293 | 0.76939 |
| FMI:No Chronic pain | -0.217 | 0.11 | -1.971 | 0.0487 |
| FMI:Chronic pain | -0.186 | 0.051 | -3.627 | 0.00029 |
|  |  |  |  |  |
| **Fully-adjusted model** | |  |  |  |
| (Intercept) | 44.62 | 0.988 | 45.148 | 0 |
| Chronic Pain | -0.416 | 0.349 | -1.192 | 0.23333 |
| Male Sex | 2.248 | 0.297 | 7.564 | 0 |
| Baseline Age | 1.218 | 0.02 | 60.397 | 0 |
| University Degree | 1.76 | 0.294 | 5.995 | 0 |
| TDI | -0.063 | 0.054 | -1.167 | 0.24341 |
| White ethnicity | 3.655 | 0.938 | 3.896 | 0.0001 |
| BMI | -0.191 | 0.033 | -5.74 | 0 |
| Current smoker | -0.204 | 0.884 | -0.231 | 0.81755 |
| poly(fup_cog0_eop, 2)1 | -524.124 | 27.307 | -19.194 | 0 |
| poly(fup_cog0_eop, 2)2 | -9.236 | 27.4 | -0.337 | 0.73605 |
| FMI:No Chronic pain | -0.187 | 0.11 | -1.7 | 0.08909 |
| FMI:Chronic pain | -0.117 | 0.052 | -2.246 | 0.02472 |

## Effect modification by sex

In participants with chronic pain, higher FMI scores were associated with lower performance across all three cognitive tasks, with more pronounced associations observed in males than females for the TMT and Matrix tasks (Figure S6). With additional adjustments for ethnicity, socioeconomic status, education, BMI, and tobacco use, the associations remained significant but were slightly attenuated across all tasks. These findings suggest that higher FMI scores are associated with worse cognitive performance in individuals with chronic pain, with stronger effects observed in males across cognitive tasks.

**
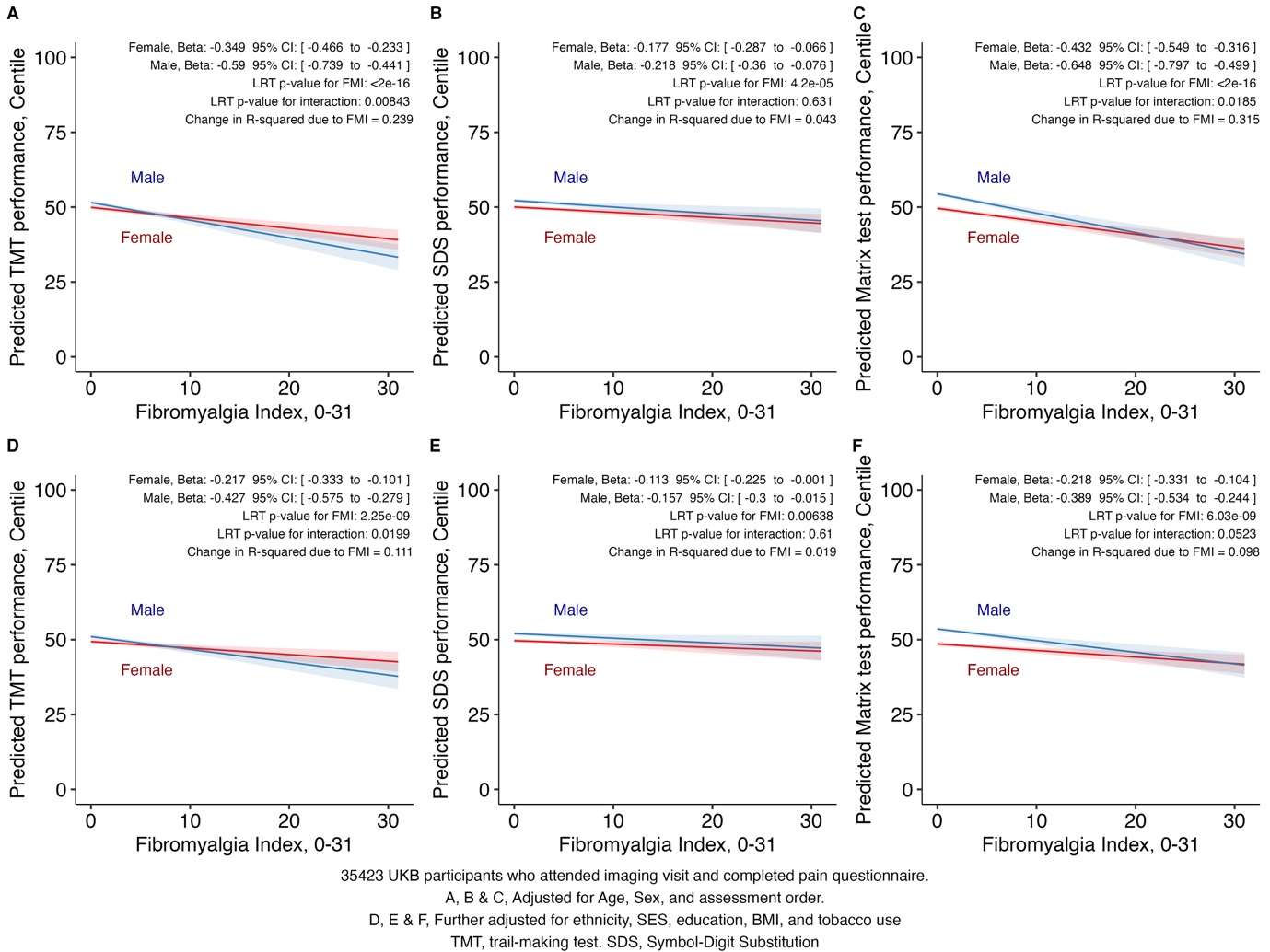
**

**Figure S6. Cross-sectional association between fibromyalgia index (FMI) and cognitive test performance by sex among participants with chronic pain.**

Panels show the predicted centile performance on the Trail Making Test (TMT, Panels A and D), Symbol-Digit Substitution (SDS, Panels B and E), and Matrix Pattern Test (Panels C and F) based on FMI scores, stratified by sex. Models in Panels A, B, and C are adjusted for age, sex, and assessment order, while models in Panels D, E, and F include further adjustments for ethnicity, socioeconomic status (SES), education, body mass index (BMI), and tobacco use. Shaded regions represent 95% confidence intervals. The negative association between FMI and cognitive performance is stronger in males across the TMT and Matrix tasks, with significant interactions between FMI and sex as indicated by likelihood ratio test (LRT) p-values for interaction.

## Assessment time discrepancies & stratification by COVID-19 period

In sensitivity analyses examining the timing of assessments and the impact of the COVID-19 pandemic on the relationship between FMI scores and executive function, consistent results were observed (Figure S7). The negative association between higher FMI scores and worse EF was stable across stratifications by the order of assessments (LRT P-interaction=0.28), the timing relative to the pain questionnaire (LRT P-interaction=0.157), and the pre- versus post-COVID-19 period (LRT P-interaction=0.0291). While a slightly stronger association was noted post-COVID, the overall relationship remained robust across all contexts, supporting the stability of the findings.


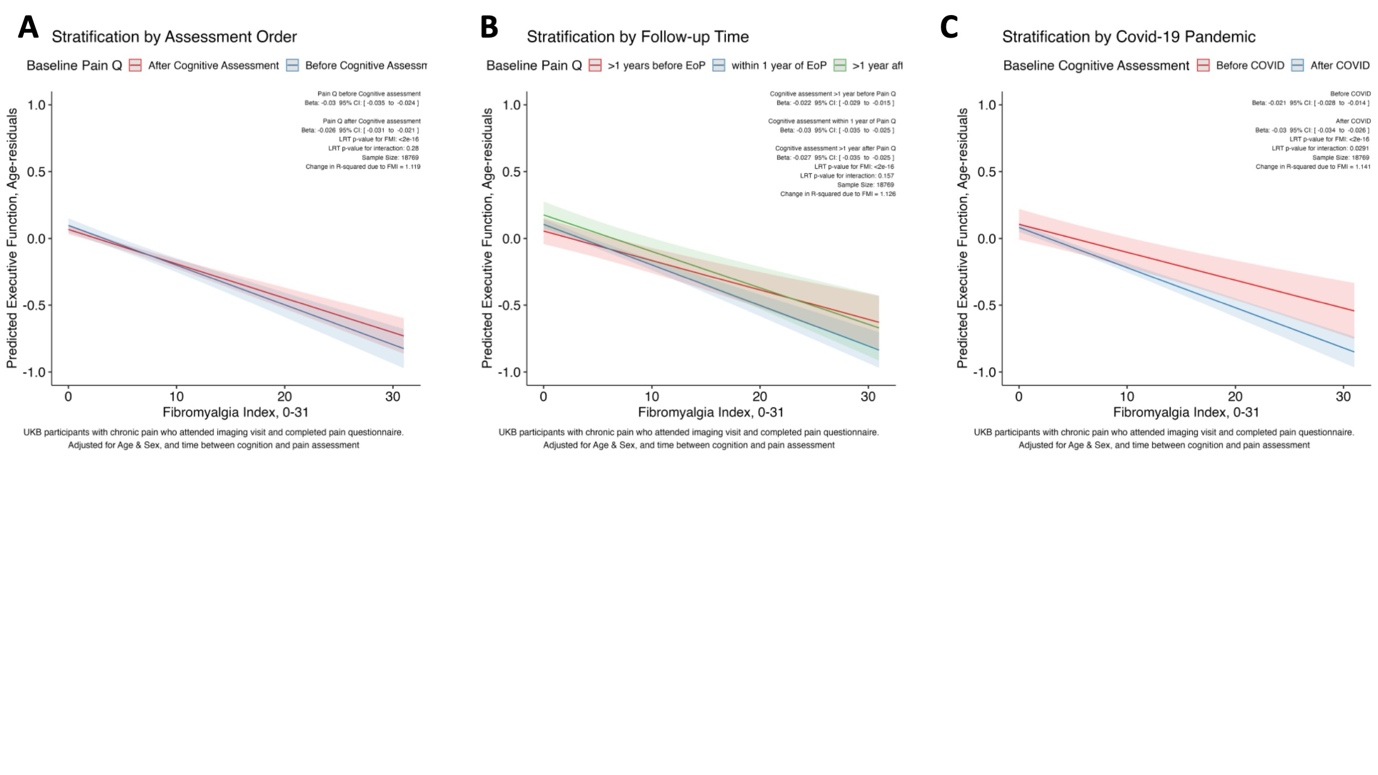


**Figure S7. Timing of cognitive assessments in relation to pain assessment and COVID-19 pandemic does not change interpretation of results.**

Sensitivity analyses of the relationship between Fibromyalgia Index (FMI) score and executive function stratified by assessment timing and external factors. Predicted executive function (age-residuals) is plotted against FMI scores for UK Biobank participants with chronic pain. Panel A shows stratification by assessment order, distinguishing whether the pain questionnaire was administered before or after cognitive testing. Panel B presents stratification by the timing of cognitive assessments relative to the pain assessment (Experience of Pain questionnaire, EoP). Panel C illustrates stratification by the timing of assessments relative to the COVID-19 pandemic. Shaded areas represent 95% confidence intervals. All models adjust for age, sex, and the time elapsed between cognition and pain assessments. The results indicate that the association between higher FMI scores and poorer EF is consistent across different assessment timings and external contexts, suggesting the robustness of this relationship.

# References

1. Cornelis MC, Wang Y, Holland T, Agarwal P, Weintraub S, Morris MC. Age and cognitive decline in the UK Biobank. *PLoS One* 2019; **14**(3): e0213948.

2. Little TD. Longitudinal structural equation modeling. New York: The Guilford Press; 2013.

3. Crawford JR, Garthwaite PH. Percentiles please: The case for expressing neuropsychological test scores and accompanying confidence limits as percentile ranks. *The Clinical Neuropsychologist* 2009; **23**(2): 193-204.

4. Widaman KF, Ferrer E, Conger RD. Factorial Invariance Within Longitudinal Structural Equation Models: Measuring the Same Construct Across Time. *Child Development Perspectives* 2010; **4**(1): 10-8.

5. Cheung GW, Rensvold RB. Evaluating goodness-of-fit indexes for testing measurement invariance. *Structural equation modeling* 2002; **9**(2): 233-55.

6. Chen FF. Sensitivity of goodness of fit indexes to lack of measurement invariance. *Structural equation modeling: a multidisciplinary journal* 2007; **14**(3): 464-504.

7. Rutkowski L, Svetina D. Assessing the hypothesis of measurement invariance in the context of large-scale international surveys. *Educational and psychological measurement* 2014; **74**(1): 31-57.
